# Central Obesity Disrupts Brain Network Organization in Aging via Metabolic and Structural Pathways

**DOI:** 10.14336/AD.2025.0887

**Published:** 2026-10-16

**Authors:** Marina Fernandez-Alvarez, Karel M. Lopez-Vilaret, Jose L. Cantero, Mercedes Atienza

**Affiliations:** ^1^Laboratory of Functional Neuroscience, Pablo de Olavide University, Seville, Spain.; ^2^CIBER de Enfermedades Neurodegenerativas (CIBERNED), Instituto de Salud Carlos III, Madrid, Spain.; ^3^Department of Psychology, Carl von Ossietzky University of Oldenburg, Germany

**Keywords:** network segregation, central obesity, overall obesity, insulin resistance, intracortical myelin, glucose PET imaging, amyloid-beta PET

## Abstract

Obesity is a recognized risk factor for age-related cognitive decline, with central (abdominal) obesity posing a particular strong threat to brain health. In a cross-sectional study of 89 cognitively healthy adults (52-79 years, mean 65.7 ± 6.4; 58 women), we compared the effects of central versus overall obesity on brain connectivity measured with resting-state fMRI. We focused on network segregation, an index of functional specialization that captures the balance between connections within and across large-scale brain networks. Central obesity, but not overall obesity, was associated with reduced segregation in associative and sensorimotor networks, even after adjusting for overall obesity, highlighting the role of abdominal fat accumulation. To explore underlying mechanisms, we combined a widely used clinical index of peripheral insulin resistance (HOMA-IR) with multimodal neuroimaging, including structural MRI for cortical thickness, T1w/T2w MRI for intracortical myelin, FDG-PET for glucose metabolism, and FBB-PET for Aβ load. Mediation analyses showed that central obesity was associated with insulin resistance, which was related to alterations in intracortical myelin, cortical glucose metabolism, and cortical Aβ accumulation. These changes were collectively linked to reduced network segregation. Modeling cortical Aβ load as preceding cortical glucose metabolism further revealed stronger and more widespread network disruption, which may reflect bidirectional interactions between amyloid pathology and metabolic dysfunction. These findings describe a pattern of metabolic and structural brain changes linked to central obesity that may compromise brain functional integrity. Although causality cannot be inferred from this cross-sectional design, targeting abdominal fat and related metabolic factors could help preserve brain health and reduce cognitive vulnerability with aging.

## INTRODUCTION

Obesity is a critical global health challenge, affecting approximately 2.5 billion adults in 2022 according to the World Health Organization (WHO) (www.who.int/news-room/fact-sheets/detail/obesity-and-overweight/). Projections indicate that by 2035, over half the global population will be overweight and nearly a quarter obese, as reported by Statista (www.statista.com/statistics/1386134/number-of-overweight-or-obese-people-orldwide-forecasts/). Among the various phenotypes of obesity, central obesity—characterized by hypertrophy of white adipocytes and excessive visceral fat surrounding abdominal organs—has emerged as particularly concerning due to its profound impact on both cardiometabolic and brain health. Central obesity currently affects 41.5% of the global population, with higher prevalence among women, older adults, urban populations, and those in high-income countries [1].

Central obesity confers a greater risk for age-related cognitive decline than overall obesity, largely through its association with insulin resistance, type 2 diabetes (T2D), hypertension, and systemic inflammation [2,3]. Individuals affected by central obesity, particularly men in some cohorts, often exhibit poorer cognitive performance independent of body mass index (BMI) [4], accompanied by reductions in gray matter (GM) volume [5,6], cortical thinning [7,8], and increased white matter (WM) hyperintensity burden [9]. Cognitive and structural alterations of this kind, shaped by sex-specific patterns of fat distribution in older adults with T2D [10] and mediated by cardiometabolic and inflammatory markers [11], are all associated with heightened dementia risk [12,13]. Collectively, this evidence underscores the multifaceted interplay between metabolic dysfunction, systemic inflammation, and sex-specific biological mechanisms in driving neurodegenerative processes, emphasizing the need for targeted strategies to mitigate the cerebral consequences of abdominal adiposity in aging populations.

Structural brain alterations associated with overall obesity are increasingly recognized as a key factor in disrupting resting-state functional connectivity (FC), critical for maintaining cognitive efficiency [14]. A central mechanism underlying these disruptions appears to be a deterioration of the brain’s functional network organization, a term describing the topological arrangement and interaction of specialized neural networks across the cortex. This breakdown reduces the brain’s capacity to support specialized processing within distinct neural networks, compromising the efficiency and integration of cognitive operations. Network segregation, a quantitative measure of functional specialization reflecting the balance of within- and between-network connectivity, has been widely used as a marker of aging-related cognitive decline [15,16]. Consistent with this, obese individuals often show reduced global and local network efficiency alongside lower modularity (a proxy for reduced segregation), indicative of compromised functional network integrity, defined as the stability and efficiency of connections within and across neural systems [17]. Furthermore, obesity has been associated with a shift toward more randomized functional brain networks, characterized by diminished local segregation and increased global integration [18]. Consistent with these observations, previous research has shown that higher BMI in healthy young adults is linked to increased differentiation between unimodal and heteromodal association networks, suggesting disrupted modular architecture and hierarchical organization of the brain [19]. These alterations are particularly evident in networks critical for cognition, such as the basal ganglia and salience systems, which show obesity-related changes in dynamic FC [20].

To our knowledge, only one study has investigated differences in brain FC between individuals with abdominal obesity and those with non-abdominal obesity [21]. The findings indicate that central obesity disrupts FC, particularly within networks implicated in behaviors linked to eating disorders, such as the executive control and frontoparietal networks. However, whether these disruptions are more pronounced in central obesity compared to overall obesity remains unclear. Additionally, the mechanisms driving these changes are not yet fully understood. Insulin resistance, systemic inflammation, and neurovascular dysfunction have been proposed as potential contributors, but further research is needed to determine their specific impact on these neural networks.

Building on previous research linking both overall and central obesity to disruptions in functional brain networks, this study aims to determine whether these obesity phenotypes differentially impact functional network segregation in cognitively normal older adults. Given that central obesity is more strongly associated with insulin resistance [22], a metabolic factor shown to reduce FC in brain regions important for executive function and memory [23], we hypothesize that central obesity will exert a more pronounced effect on functional network segregation than overall obesity.

To elucidate the mechanisms through which central obesity may impair functional network segregation, we focus on insulin resistance as a primary biological pathway. Insulin resistance is a mechanistically proximal marker that integrates the effects of other cardiometabolic disturbances common in central obesity, such as systemic inflammation [24], hypertension [25], and dyslipidemia [26], allowing us to capture their shared influence on brain structure and network organization without modeling each factor separately. Evidence indicates that insulin resistance is associated not only with alterations in WM microstructure [23,27,28] but also with decreased hippocampal volume [23] and cortical thinning, at least in middle-aged women with adiposity-related insulin resistance [29]. Similarly, higher allostatic load, a marker of cumulative metabolic and physiological stress, has been linked to region-specific changes in cortical thickness depending on body-weight status [30], underscoring that multiple forms of metabolic strain can influence structural brain integrity.

To capture these effects on GM integrity, we used two complementary measures: cortical thickness, reflecting macrostructural changes, and intracortical myelin, which provides a sensitive index of microstructural integrity vulnerable to metabolic dysregulation. Prior work in adults and older populations has linked higher BMI to reductions in cortical thickness across frontal, temporal, parietal, cingulate, and insular regions [31,32]. In addition, one study specifically reported that greater visceral but not subcutaneous fat was associated with reduced cortical thickness, and that this relationship persisted after adjustment for BMI [8, see also 33]. Together, these findings suggest that both overall and central adiposity may contribute to cortical thinning, although results are not entirely consistent, with some reports of cortical thickening [34] or age-by-BMI interactions [35]. Beyond these observational findings, it has been shown that higher insulin resistance was associated with reduced cortical thickness in frontal regions in individuals with T2D, indicating that metabolic dysregulation may influence GM structure [36]. This provides a conceptual rationale for examining insulin resistance as a potential mediator of obesity-related changes in cortical GM measures.

Intracortical myelin further complements this approach, as it reflects microstructural properties that are highly relevant to functional network segregation. Unlike WM myelin, which primarily supports long-range communication across distributed networks [37,38], intracortical myelin is essential for local circuit specialization and has been systematically associated with the intrinsic organization of cortical networks [39-41]. Although evidence linking obesity to intracortical myelin is limited, reductions have been observed in individuals with higher overall adiposity [42]. Whether central obesity exerts similar effects, and whether such effects are mediated by insulin resistance, remains unknown, making intracortical myelin a particularly suitable marker for investigating obesity-related disruptions in network segregation.

We hypothesize that insulin resistance will exert a stronger influence on intracortical myelin than on cortical thickness. Intracortical myelin is a metabolically active and functionally relevant component of the cerebral cortex, whose content can be reliably mapped using T1w/T2w and diffusion MRI techniques [43]. Although most mechanistic evidence comes from WM, where oligodendroglial fatty acid metabolism provides an energy reserve and protects myelin under conditions of limited glucose [44], the lower density of myelinated axons in cortical GM suggests that similar mechanisms may render intracortical myelin particularly sensitive to metabolic stress. Moreover, the myelin-axon interface appears especially vulnerable to disruptions in metabolic and neuroglial signaling, as evidenced in AD [45]. Intracortical myelin also correlates with cognitive decline among cognitively normal individuals [46], further highlighting its functional relevance and its potential as a sensitive marker for detecting obesity- and insulin resistance-related alterations in cortical networks before gross cortical atrophy occurs [42]. Consistent with this, we previously demonstrated that insulin resistance was more strongly associated with changes in intracortical myelin than with cortical thickness, affecting a greater number of cortical regions across two independent samples distinct from the one analyzed here [47]. Together, these lines of evidence support the notion that intracortical myelin is a particularly relevant substrate through which insulin resistance may compromise cortical integrity.

Downstream of structural alterations in intracortical myelin and cortical networks, impaired insulin signaling is expected to disrupt local cerebral glucose metabolism, which is critical for neuronal function and network stability. Evidence from aging studies [48] shows that reductions in regional cerebral glucose metabolism in older adults persist even after accounting for cortical thickness, suggesting a relationship between GM changes and glucose metabolism that is not strictly unidirectional. Complementing this, it has been reported that individuals with insulin resistance exhibit decreased glucose uptake in brain regions critical for executive function, sensory integration, and attention [23]. This pattern highlights a potential mechanism by which metabolic dysfunction may impair functional network organization. Importantly, chronic reductions in glucose availability may not only compromise synaptic activity but also interfere with the clearance of amyloid-beta (Aβ), promoting its accumulation [49]. Conversely, Aβ deposition itself can further disrupt glucose utilization and synaptic activity, generating a self-reinforcing, bidirectional cascade [50]. Through this framework, microstructural damage induced by insulin resistance may both directly compromise cortical networks and exacerbate protein mismanagement, amplifying the deleterious effects of central obesity on network segregation.

## MATERIALS AND METHODS

### Participants

This retrospective cross-sectional study included 89 adults aged 52-79 years (65.7 ± 6.4 years, 58 women). Participants were recruited from senior citizen associations, health-screening programs, and hospital outpatient services, with data collection conducted between 2021 and 2024. Inclusion criteria were: age over 50 and under 80 (to capture interindividual variability in brain and metabolic profiles during the transition from midlife to older adulthood); MMSE score ≥ 25; CDR score of 0; functional independence as assessed by the Spanish version of the Interview for Deterioration in Daily Living Activities [51]; and normal cognition based on age- and education-adjusted neuropsychological scores (for details see section 2.2). Exclusion criteria included MRI contraindications, current or past neurological or psychiatric disorders, use of psychoactive or cognition-affecting medications, and significant depressive symptoms (score ≤ 5 on the short form of the Geriatric Depression Scale [52]). Participants with severe medical conditions were also excluded, including cardiovascular diseases (e.g., recent myocardial infarction, unstable angina), severe respiratory or hepatic disorders, renal failure, active cancer, and autoimmune or infectious diseases. Metabolic conditions such as T2D, dyslipidemia, hypertension, and metabolic syndrome were allowed, as their impact on brain function and cognition was investigated indirectly through insulin resistance as an integrative marker of metabolic and vascular alterations. Eligibility was confirmed through a standardized interview by trained personnel to ensure adherence to criteria and minimize bias. The study was approved by the Ethics Committee of the Junta de Andalucía, and all participants provided informed consent.

### Neuropsychological assessment

Participants underwent a comprehensive neuro-psychological assessment to determine cognitive status, with scores interpreted according to age- and education-adjusted norms. The evaluation spanned several cognitive domains. Episodic memory was assessed using the Memory Binding Test [53], and associative memory with the Spanish Face-Name Associative Memory Exam [54]. Attention was evaluated through selective and sustained attention indices of the D2 Attention Test [55], while processing speed was measured with the Trail Making Test Part A (TMT-A) [56] and the total responses (TR) of the D2. Working memory was assessed with the letter-number sequencing subtest of the WAIS-III, and mental flexibility with the Trail Making Test Part B (TMT-B) [56]. Mental flexibility, as a component of executive functioning, was evaluated with the Trail Making Test Part B (TMT-B) and both semantic and phonological verbal fluency tasks [57]. Planning was measured with the Tower of London [58], and language abilities using the short form of the Boston Naming Test [59]. All tests were administered by a trained neuropsychologist following standardized procedures.

### Anthropometric, cardiovascular, and biochemical measurements

Trained personnel assessed participants’ anthropometric characteristics, starting with height, recorded with a stadiometer, and body weight, which was measured using a Tanita MC780 MA eight-electrode bioelectrical impedance scale (Tanita Corporation, Tokyo, Japan), from which BMI (kg/m²) was calculated to evaluate overall obesity. Waist circumference (WC), used to assess central obesity, was measured at the midpoint between the lowest rib and the iliac crest. To ensure accuracy, this measurement was taken three times, and the average value was used for analysis. The choice of WC over waist-to-hip ratio (WHR) was based on several considerations. WC provides an absolute measure of abdominal fat, which is strongly associated with insulin resistance, systemic inflammation, and metabolic dysregulation [60,61]. While WHR reflects relative fat distribution, WC has been shown to better predict metabolic dysregulation and associated vascular risk, which are key mechanisms affecting brain function [62]. Additionally, WC allows for the application of sex-specific cutoffs, adjusting for differences in fat distribution between men and women [63].

Blood pressure was measured with an automated sphygmomanometer after a 10-minute rest. Morning blood samples were collected by trained staff via venipuncture after overnight fasting, processed within 2 hours, and immediately stored at -80°C until analysis. Glucose was measured using an A15 Random Access Analyzer® (Biosystems, Spain). Serum insulin levels were quantified via Quantikine ELISA kits (R&D Systems, Minneapolis, MN, USA) in duplicate, averaging the two replicas for statistical analyses. Insulin resistance was calculated using the Homeostatic Model Assessment (HOMA-IR) as (fasting insulin [mU/l] × fasting glucose [mmol/l]) / 22.5 [64]. Insulin concentrations reported in SI units were converted to mU/l by dividing by 6 [65].

### APOE genotyping

Genomic DNA was obtained with the salting-out method [66]. APOE genotyping was performed with pre-designed TaqMan® SNP assays (rs429358 and rs7412, Applied Biosystems™, Thermo Fisher Scientific).

### MRI data acquisition and preprocessing

Brain imaging was performed in a 3T Philips Ingenia CX MRI scanner equipped with a 32-channel head coil. Prior to scanning, participants were carefully screened to ensure no contraindications to MRI, and they received comprehensive instructions to ensure full understanding of the procedure. Foam padding minimized head motion. The imaging protocol included the following sequences: i) a 3D T1w Magnetization-Prepared Rapid Gradient Echo (MP-RAGE) sequence in the sagittal plane (TR = 2600 ms, TE = 4.7 ms, flip angle = 9°, voxel size = 0.65 mm³); ii) a 3D T2w VISTA Turbo Spin Echo sequence in the sagittal plane (TR = 2500 ms, TE = 251 ms, flip angle = 90°, voxel size = 0.65 mm³); and iii) a T2w Fast Field Echo sequence with BOLD echo-planar imaging (EPI) in the axial plane (TR = 2000 ms, TE = 30 ms, flip angle = 80°, voxel size = 3 mm³, 1 mm slice gap). Physiological data including pulse and respiratory rates were simultaneously recorded via a pulse oximeter and a pneumatic belt around the abdomen. Before starting the EPI sequence, participants were instructed to remain still, keep their eyes closed, and avoid falling asleep. A total of 250 EPI scans were collected, preceded by 4 dummy volumes for spin equilibrium. A B1 calibration scan was performed before acquiring the EPI data to optimize magnetic field homogeneity. After each sequence, images were inspected to confirm they were artifact-free.

Structural MRI preprocessing was performed with Freesurfer v6.0 (https://surfer.nmr.mgh.harvard.edu/). Cortical thickness was calculated as the shortest distance between the pial surface and the GM-WM boundary. To assess intracortical myelin, T1w and T2w images were co-registered, intensity inhomogeneities were corrected, and the T1w/T2w ratio map was created. Detailed information on MRI preprocessing, cortical thickness estimation, and T1w/T2w ratio map generation is provided in Appendix 1 of the Supplementary Material.

Resting-state fMRI preprocessing was performed using AFNI v20.3.03 (https://afni.nimh.nih.gov/). Data were band-pass filtered (0.01-0.1 Hz), motion-related outliers were excluded, and physiological noise correction was applied (detailed information is provided in Appendix 2 of the Supplementary Material).

### PET data acquisition and preprocessing

PET imaging with 2-[18F]fluoro-2-deoxy-D-glucose (FDG) and Florbetaben (FBB) was used to assess cortical glucose metabolism and cortical FBB binding to Aβ deposits, respectively, in two separate sessions after 8 hours of fasting for the FDG-PET scan. Standardized uptake value ratios (SUVR) were calculated using mean cerebellar GM as the reference for FBB-PET and using pons as the reference for FDG-PET (detailed information is provided in Appendix 3 of the Supplementary Material).

### Computation of network segregation

The network segregation index was calculated as follows [16]:

(1) $$\mathrm{Network segregation}=\frac{{\overset{-}{Z}}_{W}-{\overset{-}{Z}}_{B}}{{\overset{-}{Z}}_{W}}$$

where ${\overset{-}{Z}}_{W}$is the mean Fisher *z*-transformed *r* between nodes within the same cortical network (within-network FC) and ${\overset{-}{Z}}_{B}$is the mean Fisher *z*-transformed *r* between nodes of one cortical network and all nodes in other cortical networks (between-network FC). Negative z-values were excluded.

Resting-state network segregation used a surface-based parcellation comprising 333 parcels across 12 cortical networks [67], defined by high-dimensional cortical features and BOLD signal characteristics (see Supplementary Figure 1 in Appendix 4). This approach accurately captures the brain's topological organization, essential for evaluating network segregation.

To assess contributions of different brain imaging modalities to network segregation, modality-specific data were averaged across cortical parcels [47]. Partialized within- and between-network FC were calculated as follows:

(2) $$y_{i=}=\beta_{0}+\beta_{1}R_{i}+e_{i}(2)$$

where yrepresents the outcome (either ${\overset{-}{Z}}_{W}$ or ${\overset{-}{Z}}_{B}$), Rthe imaging modality, βthe model parameter, ethe residual, and ithe parcel. Partialized FC ( ${\mathrm{PFC}}_{i}$) was calculated as:

(3) $${\mathrm{PFC}}_{i}=\sum_{i=1}^{n}\left(y_{i}-\beta_{1}R_{i}\right)(3)$$

Partialized within-network FC ( ${\overset{-}{Z}}_{W}$) and between-network FC ( ${\overset{-}{Z}}_{B}$) were obtained by averaging the z-transformed ${\mathrm{PFC}}_{i}$ across parcels within each cortical network, and the segregation index was computed using the equation (1).

### Coupling between brain imaging modalities at the individual level

Local associations between brain imaging modalities were evaluated via inter-modal coupling analysis [68]. This approach captures the localized association between two cortical maps near a given vertex. For each vertex, a 15-mm FWHM neighborhood was defined, and locally weighted regression was applied, with one modality (e.g., FBB-PET) as the dependent variable and the other (e.g., FDG-PET) as the fixed regressor. In some cases, a third imaging modality (e.g., T1w/T2w) was included as a covariate.

## Statistical analysis

### Effect of overall and central obesity on brain network segregation

Overall obesity was defined as a categorical variable based on BMI-derived classifications: normal weight (18.5-24.9 kg/m²), overweight (25-29.9 kg/m²), and obesity (≥ 30 kg/m²), following widely accepted WHO criteria (www.who.int/news-room/fact-sheets/detail/ obesity-and-overweight/). Central obesity was defined dichotomously (absent vs. present) using sex-specific WC thresholds (≥ 94.5 cm for men; ≥ 89.5 cm for women) validated for the Spanish population [69].

Statistical analyses were performed using MATLAB R2023b (MathWorks, USA). Linear regression models were used to examine the effects of obesity on brain network segregation, adjusting for age, sex, years of education, APOE4 status, and the alternate measure of adiposity. Education and APOE4 were included to account for differences in cognitive reserve and genetic influences on brain structure and function. The alternate adiposity variable was entered as a categorical covariate to control for potential non-linear effects and confounding between adiposity measures. For robustness, analyses were also repeated treating this variable as continuous, which yielded consistent results. To prevent multicollinearity, BMI and WC were not included simultaneously as continuous predictors. Because sex influences fat distribution, endocrine function, and cardiometabolic responses, we additionally explored whether sex moderated the associations of overall and central obesity with network segregation by including obesity × sex interaction terms. These interaction analyses were performed on an exploratory basis because the sample size may not provide sufficient power to reliably identify sex-specific differences.

The Cook's distance method was used to identify outliers; however, as the results remained consistent after their removal, all participants were retained in the final analysis. Fixed- and mixed-effects models were fitted using *fitlm* and *fitlme*, respectively. Local standardized effect sizes were estimated whenever significant effects of the predictor variable on the outcome were observed. Main effects and interactions between categorical variables were quantified via Cohen’s f² [70], while interaction effects including a continuous variable were assessed by calculating δ (categorical × continuous) and ρ (continuous × continuous) using Bodner’s approximation [71]. Effect sizes were classified as small (S), moderate (M), or large (L), indicated as superscripts. Additionally, 95% bias-corrected and accelerated (BCa) bootstrap confidence intervals (95% CI) were computed for all effect size estimates to assess their precision.

### Mediation analysis

To investigate potential mechanisms linking obesity to network segregation in sensorimotor (SMN) and associative (ASN) systems, we conducted a series of mediation analyses with values averaged across the networks shown in Supplementary Figure 1 (Appendix 4). We assessed the individual contribution of candidate mediators using simple mediation models, while dual mediation models explored potential sequential indirect pathways and multiple mediator models examined the joint effects of additional mediators. Covariates in all models included age, sex, education, APOE4 status, and the alternative obesity measure. Additionally, exploratory analyses evaluated whether the associations between obesity and each mediator were moderated by sex.

Rather than relying solely on traditional bootstrapping methods, we adopted a stepwise mediation approach to accommodate the complexity of our data. While conventional bootstrapping is typically applied to summary scores and provides confidence intervals through resampling, our stepwise approach enables vertex-wise evaluation of imaging measures, sequential testing of single and multiple mediators, and structured confirmation of mediation effects. This strategy allows for a clear interpretation of both individual and combined contributions of candidate mediators while appropriately controlling for covariates and potential moderators. Detailed procedures for each type of mediation model are described below.

### Simple mediation analysis

When HOMA-IR was considered as a potential mediator, a standard mediation analysis framework was followed. The effect of obesity (overall or central) on HOMA-IR (path A) was tested, adjusting for covariates such as the alternative obesity variable, age, sex, years of education, and APOE4 genotype. The impact of HOMA-IR on the segregation of the SMN and ASN (path B) was then assessed, controlling for the same covariates. Sex was also explored as a potential moderator in both steps of the mediation model, treated as an exploratory analysis. Finally, the direct effect of obesity on network segregation was examined to determine whether it became non-significant when HOMA-IR was included. Mediation was estimated using the product of coefficients method (path A × path B), with significance determined by bootstrapped confidence intervals.

For imaging-based mediators, a three-step approach was applied. First, we tested the association between obesity and each imaging modality at the cortical surface on a vertex-by-vertex basis, including interactions with sex and adjusting for covariates. Surface-based statistical analyses were conducted using the SurfStat package (www.math.mcgill.ca/keith/surfstat/). Results were corrected for multiple comparisons using a hierarchical procedure that first controls the family-wise error rate (FWER) at the cluster level by applying random field theory to smoothed statistical maps, followed by false discovery rate (FDR) correction at the vertex level within each cluster on unsmoothed maps [72]. Vertex-level significance was set at p < 0.001, and cluster-level significance at p < 0.05. Surviving clusters were anatomically localized using peak coordinates from the Desikan-Killiany atlas [73]. Second, the imaging modality was evaluated as a predictor of network segregation, adjusting for covariates and the obesity variable, by comparing segregation values before and after partialling out connectivity based on the imaging modality using mixed-effects models. Third, mediation was confirmed by testing whether the effect of obesity on network segregation became non-significant after accounting for the imaging modality.

### Dual mediator analysis

Sequential mediation analyses were conducted to evaluate whether HOMA-IR and an imaging modality jointly mediated the relationship between obesity and network segregation. This approach involved three steps: First, we demonstrated a significant effect of HOMA-IR on the imaging modality, assessed vertex-wise on the cortical surface and adjusted for the two obesity variables and other covariates. Next, we assessed network segregation differences before and after partialling out the imaging modality, while controlling for the obesity variables, HOMA-IR, and covariates. Finally, we confirmed mediation by showing that the direct effect of obesity on network segregation became non-significant after accounting for HOMA-IR, the imaging modality, and all covariates.

### Multiple mediator analysis

For more complex sequential models involving additional imaging modalities, the coupling between two imaging modalities was first computed, with significantly coupled regions identified using one-sample t-tests. The effect of HOMA-IR on this coupling was then tested, followed by an evaluation of differences in network segregation before and after partialling out the residuals of the coupling while adjusting for all variables in the model. Mediation was ultimately confirmed if the direct effect of obesity on network segregation became non-significant after accounting for obesity variables, the imaging coupling, HOMA-IR, and common covariates.

**Table 1:** Demographic and metabolic characteristics by overall and central obesity for the whole sample (male = 31; female = 58).

Variable	Overall obesity			Central obesity	
	Normal	Overweight	Obesity	No	Yes
**Participants**	30	37	22	49	40
**Females (%)**	22 (73.3)	22 (59.5)	14 (63.6)	37 (75.5)	21 (52.5)
**Age**	66.3 ± 6.5	65.2 ± 5.7	65.9 ± 7.3	66.2 ± 6.1	65.1 ± 6.7
**Education years**	11.3 ± 5.3	12.0 ± 4.6	9.1 ± 4.4	11.9 ± 5.4	10.1 ± 4.1
**APOE4 (%)**	6 (20.0)	11 (29.7)	4 (18.2)	10 (20.4)	11 (27.5)
BMI	22.0 ± 1.6	27.0 ± 1.4	33.1 ± 3.0	23.9 ± 2.8	30.5 ± 3.8
**Waist circumference (cm)**	80.1 ± 8.0	90.8 ± 7.9	104.3 ± 9.4	81.8 ± 7.2	101.2 ± 8.3
**Glucose (mmol/l)**	4.9 ± 0.6	5.2 ± 0.9	5.4 ± 0.7	4.9 ± 0.5	5.5 ± 0.9
**Insulin (mU/l)**	4.8 ± 2.3	7.5 ± 3.9	13.1 ± 6.6	5.2 ± 2.5	11.3 ± 6.0
**HOMA-IR**	1.1 ± 0.6	1.8 ± 1.2	3.2 ± 1.7	1.2 ± 0.6	2.9 ± 1.7
**HbA1c (%)**	5.4 ± 0.5	5.5 ± 0.7	5.7 ± 0.8	5.4 ± 0.5	5.6 ± 0.8
**Hypertension (%)**	12 (40.0)	18 (48.6)	13 (59.0)	21 (42.8)	22 (55.0)
**Dyslipidemia (%)**	5 (16.7)	4 (10.8)	6 (27.3)	8 (16.3)	7 (17.5)
**Type 2 diabetes (%)**	1 (3.3)	2 (5.4)	3 (13.6)	1 (2.0)	0 (0)
**Metabolic syndrome (%)**	1 (3.3)	4 (10.8)	7 (31.8)	5 (10.2)	12 (30.0)

Note. Continuous variables are shown as mean ± SD. Categorical variables are shown as n (%) within each obesity category. BMI = body mass index; HbA1c = glycoside hemoglobin

## RESULTS

### Sample characteristics

The Table 1 summarizes participants’ demographic and metabolic profiles (mean ± SD) for the overall sample (i.e. men and women combined), stratified by overall and central obesity categories. Age, education, and APOE4 carrier status did not differ across obesity categories, whereas sex differed for central obesity (OR = 2.8; 95% CI = 1.1-7.3; p = 0.025; AUC = 0.63), reflecting a higher relative likelihood of central obesity in men compared with women. Neither overall nor central obesity increased the risk of hypertension, T2D, or dyslipidemia; however, overall obesity was linked to a greater risk of metabolic syndrome relative to normal weight (OR = 13.5; 95 % CI = 1.5-120.5; p = 0.019; AUC = 0.74), and this effect remained significant after adjusting for age, sex, education years, and APOE4 (OR = 16.7, 95% CI (1.7 167.2), p = 0.017, AUC =0.78). When examining sex differences within each obesity category, BMI was higher in men than in women only within the normal-weight group, while among those with central obesity, men were younger, had more years of education, and exhibited larger WC than women. Complete sex-stratified results are provided in Supplementary Tables 1 and 2 (Appendix 5), with significant differences indicated by superscripts (*p < 0.05).

Both overall and central obesity classifications were significantly associated with BMI, WC, insulin levels, and HOMA-IR after adjusting by age, sex, education, and APOE4. Statistical details are shown in Supplementary Table 3 (Appendix 5). Fasting glucose, but not HbA1c, differed between individuals with and without central obesity, whereas neither glucose nor HbA1c showed significant differences across BMI categories.

In addition, Supplementary Table 4 (Appendix 5) presents the mean (± SD) of scores from neuropsychological tests covering multiple cognitive domains by obesity category. We found better performance of the overweight group on the Letter-Number Sequencing and TMT-B tests, and lower (better) TMT-B scores in participants with central obesity. However, all associations lost significance after FWE correction. These exploratory findings suggest that cognitive differences are minimal and unlikely to account for the obesity-related effects on network segregation reported below.

**Figure 1.**
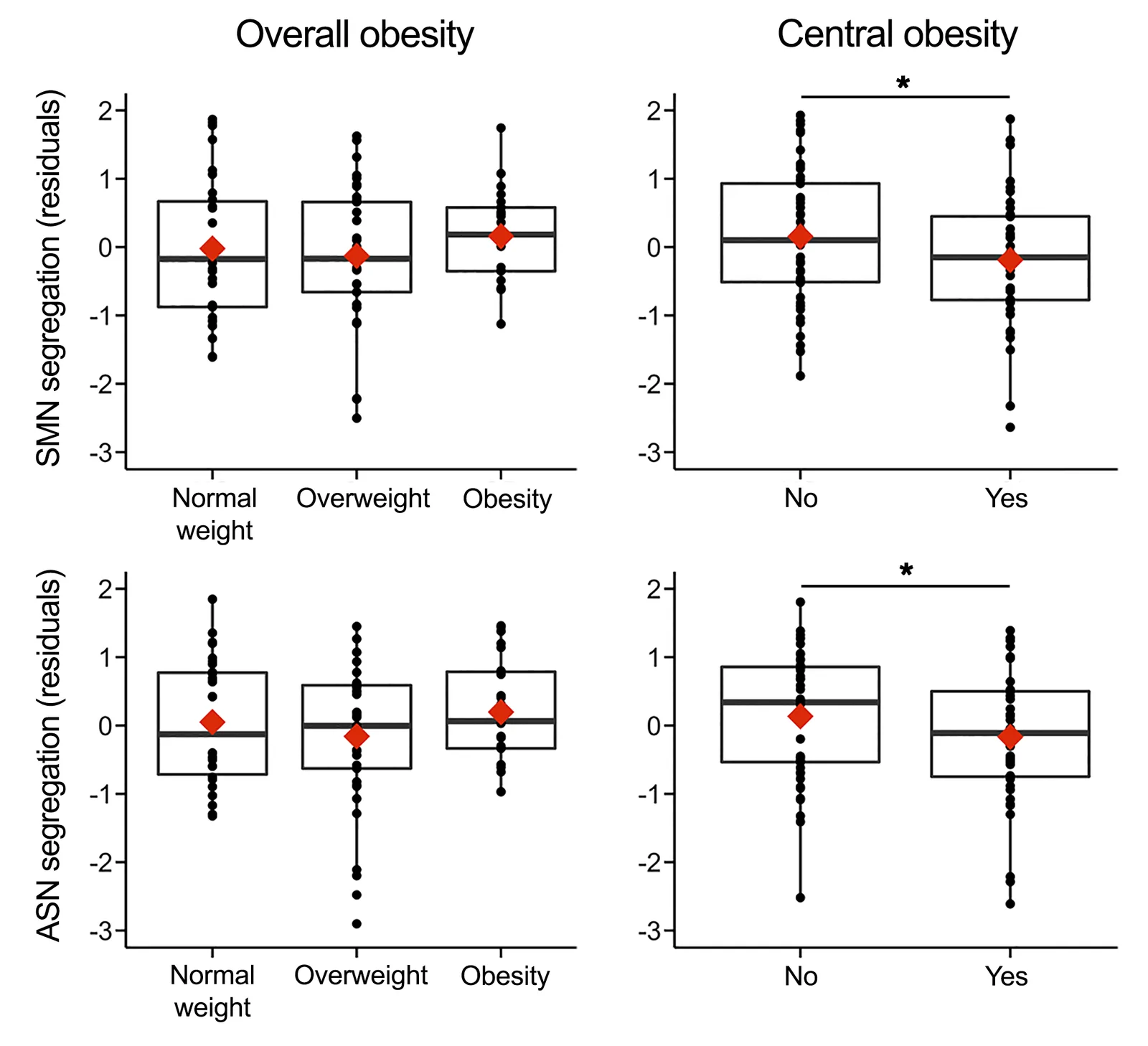
**Bar plots illustrating the effects of overall and central obesity on network segregation**. The plots on the left column show the effect of overall obesity on SMN segregation (top) and ASN segregation (bottom), while the plots on the right column depicts the effect of central obesity. The y-axis represents the residuals of segregation, adjusted for age, sex, education years, APOE4, and the alternate obesity measure. Each bar plot includes interquartile ranges (boxes), the median, the mean (red diamond), individual subject data points, and whiskers. In some cases, one or two data points fall outside the whiskers, indicating potential outliers.

### Obesity’s effect on network segregation

Linear regression analyses indicated that overall obesity had no significant impact on network segregation, whereas central obesity was significantly associated with segregation of both SMN (F_1,83_ = 4.70, p_FWE_ = 0.033, f^2^ = 0.06^S^ [0.0009 0.22]) and ASN (F_1,83_ = 6.03; p_FWE_ = 0.032, f^2^ = 0.07^S^ [0.003 0.24]). These effects remained after adjusting for common covariates as age, sex, education years, and APOE4. Further analyses showed that even after controlling for overall obesity as a categorical covariate, central obesity continued to be significantly associated with decreased segregation of both SMN (F_1,81_ = 6.54, p_FWE_ = 0.025, f^2^ = 0.08^S^ [0.002 0.29]) and ASN (F_1,81_ = 5.17, p_FWE_ = 0.026, f^2^ = 0.07^S^ [0.0008 0.25]). Figure 1 shows the associations between overall and central obesity and network segregation, adjusted for all covariates. We identified 4 to 7 potential outliers, but results did not change when they were removed, so we retained them in subsequent analyses.

To further rule out the possibility that central obesity associations merely reflect differences in overall obesity, we performed additional sensitivity analyses. When adjusting the models for BMI as a continuous covariate, the relationship between central obesity and network segregation remained significant for both SMN (F_1,82_ = 8.87, p_FWE_ = 0.008, f^2^ = 0.08^S^ [0.003 0.29]) and ASN (F_1,82_ = 5.9, p_FWE_ = 0.017, f^2^ = 0.06^S^ [0.0009 0.25]). We also repeated the analyses using BMI as a binary categorical variable (low vs. high BMI), created to match the sample distribution of the central obesity groups. This binarization helped balance group sizes and reduce multicollinearity, facilitating a more direct comparison between groups with similar characteristics. In this model, the association of central obesity with SMN segregation remained significant (F_1,82_ = 5.45, p_FWE_ = 0.044, f^2^ = 0.07^S^ [0.002 0.25]), whereas the association of ASN with segregation did not reach significance (F_1,82_ = 1.99, p_FWE_ = 0.16, f^2^ = 0.02^S^ [0.00002 0.16]). These findings support the interpretation that the observed effects, particularly for SMN, are driven primarily by central obesity rather than by overall obesity. Accordingly, in the subsequent mediation analyses we included overall obesity categorized into three clinically meaningful groups as a covariate, so as to adjust for global adiposity while retaining power to detect mediation effects that may be specific to central obesity.

Given the observed sex imbalance in central obesity, we also conducted exploratory analyses to test whether sex moderated the effects of central or overall obesity on network segregation. These models were adjusted for age, education years, APOE4, and the other obesity measure. No significant sex × obesity interactions were observed for either SMN or ASN segregation (p_FWE_ > 0.9). Effect sizes were small (f^2^ < 0.12) and 95% CI included zero, indicating no meaningful moderation by sex.

### Identification of single mediators of the association between central obesity and brain network segregation

Central obesity was significantly related to HOMA-IR after adjusting for covariates, including overall obesity (F_1,81_ = 6.6, p_FWE_ = 0.012, f^2^ = 0.08^S^ [0.0009 0.31]). Specifically, individuals with central obesity had a mean HOMA-IR value that was 2.4 times higher than those without central obesity. Exploratory analyses showed that this association was not moderated by sex. Although the interaction term did not reach statistical significance (p_FWE_ > 0.2), the effect size was moderate (f^2^ = 0.13) with a 95% CI of [0.009 0.32], suggesting a possible influence that our study may have been underpowered to detect. Nevertheless, HOMA-IR showed no significant main effect on network segregation after adjusting for central obesity and covariates, indicating that it does not act as a direct mediator of this association.

Central obesity was also significantly associated with changes in cortical thickness, with individuals exhibiting central obesity showing increased cortical thickness in the right hemisphere, specifically in the middle temporal gyrus (F_11,81_ = 16.1, p_FWE_ = 0.001, f^2^ = 0.20^M^ [0.03 0.53]) and lingual gyrus (F_11,81_ = 14.9, p_FWE_ = 0.04, f^2^ = 0.18^M^ [0.04 0.45]). Further analysis examined the association of cortical thickness with network segregation while controlling for central obesity and other covariates. A mixed-effects model was employed to compare network segregation before and after adjusting the FC between nodes for the cortical thickness of the corresponding nodes. The results revealed reduced segregation in the ASN after accounting for cortical thickness in individuals with central obesity (F_1,169_ = 6.2, p_FWE_ = 0.027, f^2^ = 0.08^M^ [0.0002 0.39]). However, no such effect was observed for SMN segregation.

Despite the significant findings reported above, cortical thickness did not act as a mediator. When assessing the association of central obesity with network segregation while isolating the contribution of cortical thickness, it continued to be significantly related to both SMN (F_1,81_ = 5.9, p_FWE_ = 0.034, f^2^ = 0.07^S^, [0.001 0.29]) and ASN (F_1,81_ = 5.8, p_FWE_ = 0.018, f^2^ = 0.07^S^, [0.0009 0.31]).

In summary, central obesity was associated with elevated HOMA-IR and increased cortical thickness, yet neither factor acted as a direct mediator of the relationship between central adiposity and network segregation once covariates were considered. Central obesity showed no significant effects on intracortical myelin, cortical glucose metabolism, or Aβ load, and exploratory analyses also indicated that these patterns were largely consistent across men and women. To provide a comprehensive overview, Supplementary Table 5 (Appendix 5) reports descriptive statistics for cortical thickness, intracortical myelin, FDG-PET, FBB-PET, and network segregation stratified by sex and central obesity group. Despite the absence of significant sex interactions, HOMA-IR may still influence downstream neurobiological processes, suggesting potential sequential mediation pathways. In the following section, we examined these pathways by analyzing the associations between HOMA-IR and multiple brain imaging modalities, while adjusting for both central and overall obesity as well as common covariates.

**Figure 2.**
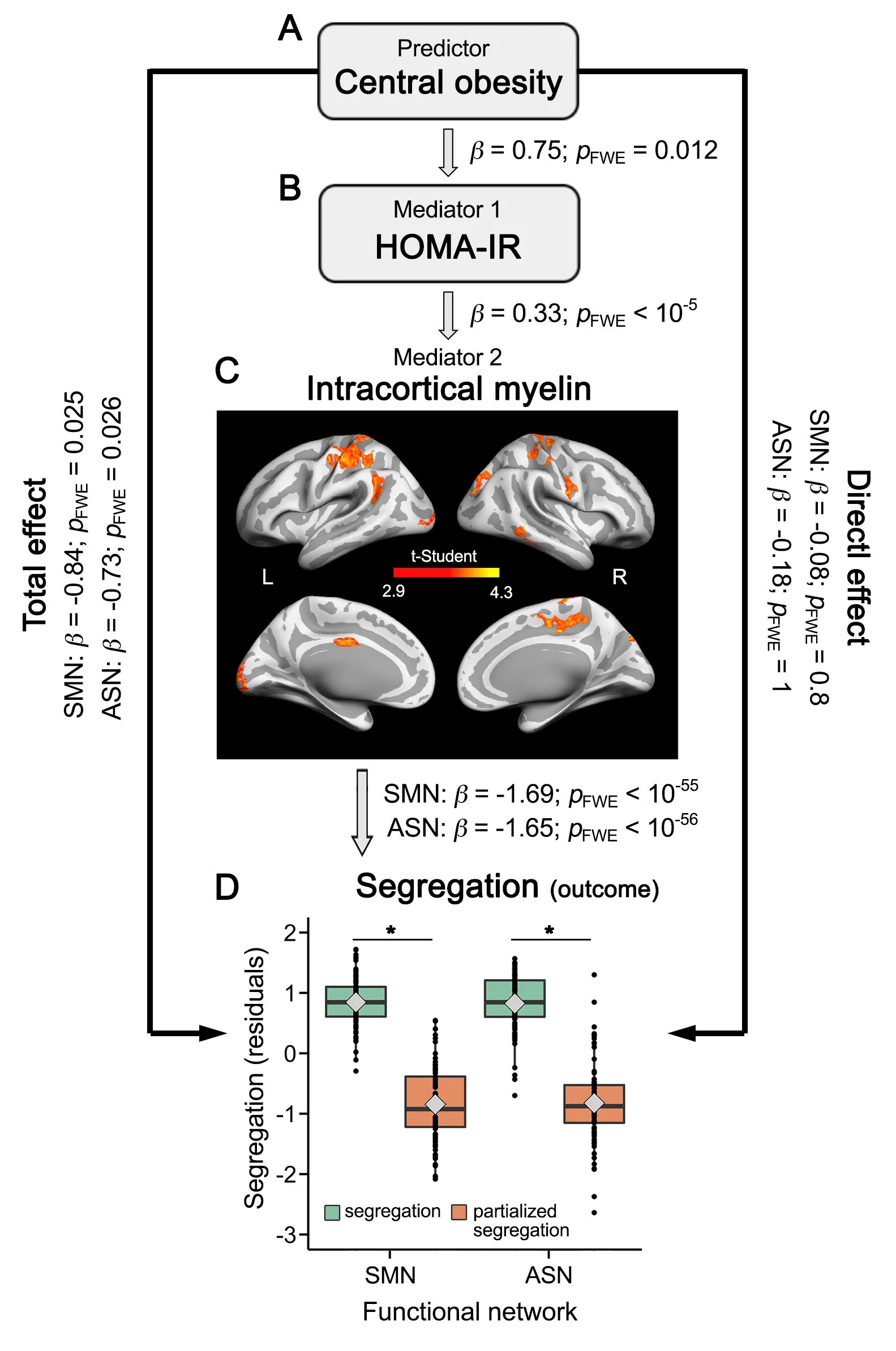
**Mediation model examining the relationships among central obesity, HOMA-IR, intracortical myelin, and network segregation**. (**A**) Effect of central obesity (predictor) on HOMA-IR (mediator 1) after adjusting for covariates such as age, sex, education years, APOE4, and overall obesity. Standardized betas (β) and FWE-corrected p-values (p_FWE_) are shown. The total effect from central obesity to network segregation is represented by an arrow on the left side, while the direct effect is depicted by a mirrored arrow on the right side. (**B**) Effect of HOMA-IR (mediator 1) on intracortical myelin (mediator 2) after adjusting for central obesity and covariates with standardized β and p_FWE_ indicated. C) Lateral and medial views of the left and right hemispheres highlight brain regions where intracortical myelin is significantly associated with HOMA-IR (p_FWE_ < 0.05) after the corresponding adjustment. Warmer colors indicate regions where higher HOMA-IR corresponds to increased intracortical myelin. Detailed statistical outcomes, including the anatomical locations of significant clusters, are provided in Table 2. (**D**) Network segregation for sensorimotor (SMN) and associative networks (ASN), shown before and after controlling for intracortical myelin, while also adjusting for central obesity, HOMA-IR, and covariates. Significant differences are marked with asterisks (p_FWE_ < 0.05). Bar plots show individual subject data points, medians, means (grey diamonds), interquartile ranges (boxes), and whiskers; outliers outside the whiskers are indicated when present.

### Dual mediators of the association between central obesity and network segregation

Regression analyses identified a significant association of HOMA-IR with GM macro- and microstructure, but no significant associations were observed with cortical metabolic activity or Aβ load. In the right hemisphere, higher HOMA-IR levels were related to thickening in the middle temporal gyrus (F_12,80_ = 11.4, p_FWE_ = 0.033, f^2^ = 0.14^S^ [0.02 0.40]) and precentral gyrus (F_12,80_ = 12.1, p_FWE_ = 0.047, f^2^ = 0.15^M^ [0.02 0.41]). Regression analyses also showed a significant positive association between HOMA-IR and intracortical myelin across widespread cortical areas, including motor, visual, memory, and higher-order cognitive regions (Fig. 2C). Detailed statistical outcomes are in Table 2.

**Table 2:** Association between HOMA-IR and intracortical myelin adjusted by central obesity and covariates (age, sex, education years, APOE4, and overall obesity).

Cortical region	MNI coordinates	R^2^	F_12,80_	*p*	Size effect (f^2^)	*95% CI*
**L postcentral gyrus**	-50 -24 56	0.17	18.0	10^-5^	0.23^M^	0.05 - 0.55
**L lateral-occipital gyrus**	-3 -98 4	0.14	14.7	10^-5^	0.18^M^	0.05 - 0.39
**L posterior cingulate**	-5 -6 30	0.13	12.6	0.0003	0.16^M^	0.03 - 0.38
**L supramarginal gyrus**	-53 -52 31	0.13	12.6	0.04	0.16^M^	0.03 - 0.40
**R precentral gyrus**	23 -25 60	0.13	13.6	10^-5^	0.17^M^	0.04 - 0.38
**R postcentral gyrus**	50 -8 27	0.13	13.3	0.004	0.17^M^	0.05 - 0.36
**R paracentral gyrus**	15 -23 39	0.14	14.1	0.018	0.18^M^	0.03 - 0.44
**R superior parietal**	19 -81 38	0.13	13.3	0.037	0.17^M^	0.04 - 0.42
**R inferior temporal gyrus**	60 -48 -15	0.17	17.4	0.044	0.22^M^	0.06 - 0.50

Note: size effect M = moderate; L = left hemisphere; R = right hemisphere

Next, we examined whether cortical thickness and intracortical myelin were related to network segregation, controlling for central obesity, HOMA-IR, and common covariates. Adjusting FC for cortical thickness yielded a reduction in ASN segregation (F_1,168_ = 6.2, p_FWE_ = 0.027, f^2^ = 0.08^M^ [0.0001 0.39]), but no significant changes in SMN segregation. When adjusting FC for intracortical myelin (Fig. 2D), significant effects were observed in both networks, with reduced segregation in the SMN (F_1,168_ = 574.9, p_FWE_ < 10^-55^, f^2^ = 4.0^L^ [3.88 3.89]) and ASN (F_1,168_ = 608.8, p_FWE_ < 10^-56^, f^2^ =5.48^L^ [4.25 4.88]).

Finally, we investigated whether HOMA-IR and cortical GM mediated the association between central obesity and network segregation. Mediation was determined by assessing whether central obesity’s association with network segregation attenuated to non-significance after accounting for cortical GM macro- and microstructure, while adjusting for HOMA-IR and common covariates. Cortical thickness did not meet mediation criteria, as the association between central obesity and ASN segregation remained significant (F_12,80_ = 12.8, p_FWE_ = 0.005, f^2^ = 0.16^M^ [0.03 0.41]). However, mediation was confirmed for intracortical myelin, as the relationship between central obesity and segregation became non-significant for both SMN and ASN after the corresponding adjustment (Fig. 2).

### Multiple mediators: HOMA-IR → GM structure → FDG-PET

We next investigated whether the sequential relationship among HOMA-IR, cortical GM structure, and cortical glucose uptake mediated the association between central obesity and network segregation. To examine the link between GM (either cortical thickness or intracortical myelin) and cortical glucose uptake, we calculated the local coupling maps between these imaging modalities for each individual. Residuals from the coupling maps, adjusted for HOMA-IR, central obesity, and common covariates, were analyzed at the group level using one-sample *t*-tests. The cortical maps presented in Figure 3 (panels A and B) illustrate the effect sizes (Cohen's *d*) for the significant regions. While the spatial distribution of the coupling with FDG-PET was similar across both cortical thickness and intracortical myelin in medial regions, clear differences emerged in lateral regions. The positive correlation was significantly more extensive for coupling with intracortical myelin, with a larger effect size compared to cortical thickness, indicating that in lateral regions, particularly ventral areas, increased glucose uptake was associated with reduced cortical thickness and elevated intracortical myelin content.

Next, we re-computed segregation after adjusting FC for the residuals of the individual-level coupling between cortical GM structure and cortical glucose uptake, without controlling for other group-level variables. This approach assesses the association of cortical glucose consumption on network segregation while removing the contribution of cortical GM. To control for central obesity, HOMA-IR, and common covariates, we applied mixed model regression to compare segregation before and after adjusting FC by cortical GM structure and metabolism. The analysis revealed significant reductions in segregation in SMN (F_1,168_ = 593.8, p_FWE_ < 10^-56^, f^2^ = 6.78^L^ [1.9 17.4]) and ASN (F_1,168_ = 761.5, p_FWE_ < 10^-63^, f^2^ = 9.04^L^ [4.2 17.6]) after controlling the contribution of cortical thickness. A similar result was found when intracortical myelin was controlled, with reductions in both SMN (F_1,168_ = 484.8, p_FWE_ < 10^-50^, f^2^ = 5.54^L^ [0.88 17.8]) and ASN (F_1,168_ = 987.1, p_FWE_ < 10^-71^, f^2^ = 11.68^L^ [7.3 17.3]). These results are illustrated in Figure 3 (panels C and D).

The association between central obesity and segregation became non-significant for both SMN and ASN after accounting for the effects of all mediators and covariates on FC. This indicates that the sequential pathway linking HOMA-IR, cortical GM structure, and cortical glucose uptake mediates the relationship between central obesity and network segregation.

**Figure 3.**
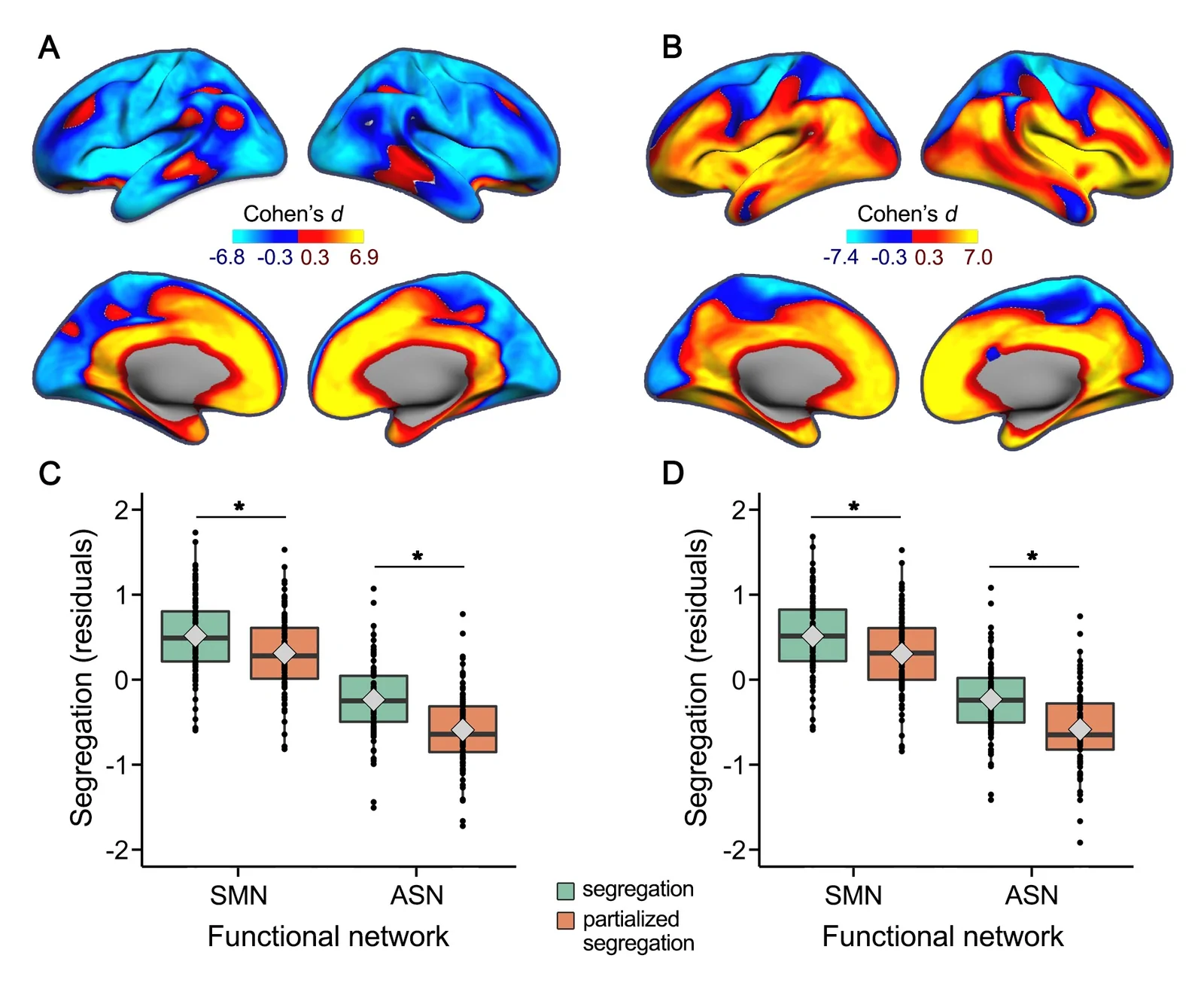
**Mediating role of GM-FDG coupling on network segregation**. (**A**) Group-level results of a one-sample t-test performed on individual coupling maps between cortical thickness and cortical glucose uptake (FDG-PET), adjusted for central obesity, HOMA-IR, and covariates (age, sex, education years, APOE4, and overall obesity). Lateral and medial views of the left and right hemispheres display effect sizes (Cohen’s d), with warm colors indicating positive associations and cool colors negative associations. (**B**) Same analysis as in A, but with intracortical myelin as the predictor. (**C**) Network segregation for sensorimotor (SMN) and associative networks (ASN) before and after adjusting FC for residuals of the individual-level coupling between cortical thickness and cortical glucose uptake, while controlling for central obesity, HOMA-IR, and covariates. Significant differences are marked with asterisks (p_FWE_ < 0.05). (**D**) Same as C, but adjusting FC for residuals of the individual-level coupling between intracortical myelin and cortical glucose uptake. Bar plots in panels C and D include individual subject data points, medians, means (grey diamonds), interquartile ranges (boxes), and whiskers; outliers outside the whiskers are indicated when present.

### Multiple mediators: HOMA-IR → GM structure → FBB-PET

The same approach was applied to assess the sequential mediation of HOMA-IR, cortical GM structure, and cortical Aβ load in the association between central obesity and network segregation. Using one-sample t-tests, we assessed the coupling between cortical GM structure and FBB-PET imaging, analyzing the residuals after controlling for HOMA-IR, central obesity, and common covariates. While significant associations were observed throughout most of the cortical mantle for both cortical thickness and intracortical myelin, the direction of the coupling was opposite in specific regions (i.e., negative for cortical thickness and positive for intracortical myelin), including the cingulate cortex, insula, somatosensory cortex, and lateral temporal cortex. The results are shown in Figure 4 (panels A and B).

Mixed model regression analysis revealed significant differences in network segregation before and after accounting for the residuals from the coupling between cortical GM structure and cortical Aβ load. Specifically, when analyzing the residuals from the coupling between cortical thickness and Aβ load, after controlling for central obesity, HOMA-IR, and common covariates, we observed a marked reduction in segregation within the SMN (F_1,168_ = 801.3, p_FWE_ < 10^-65^, f^2^ = 9.2^L^ [2.3 20.4]) and the ASN (F_1,168_ = 1447.9, p_FWE_ < 10^-83^, f^2^ = 17.2^L^ [11.0 28.0]). A similar effect was seen when accounting for intracortical myelin, resulting in significant reductions in segregation in both SMN (F_1,168_ = 1126.4, p_FWE_ < 10^-75^, f^2^ = 12.9^L^ [6.8 23.0]) and ASN (F_1,168_ = 1039.3, p_FWE_ < 10^-73^, f^2^ = 12.4^L^ [8.1 21.0]). These results are shown in Figure 4 (panels C and D).

Finally, the regression analysis of central obesity and network segregation, after controlling for all mediators and covariates, confirmed a mediating role of the pathway linking HOMA-IR with cortical GM and Aβ load, as the association between central obesity and network was no longer significant for both SMN and ASN.

**Figure 4.**
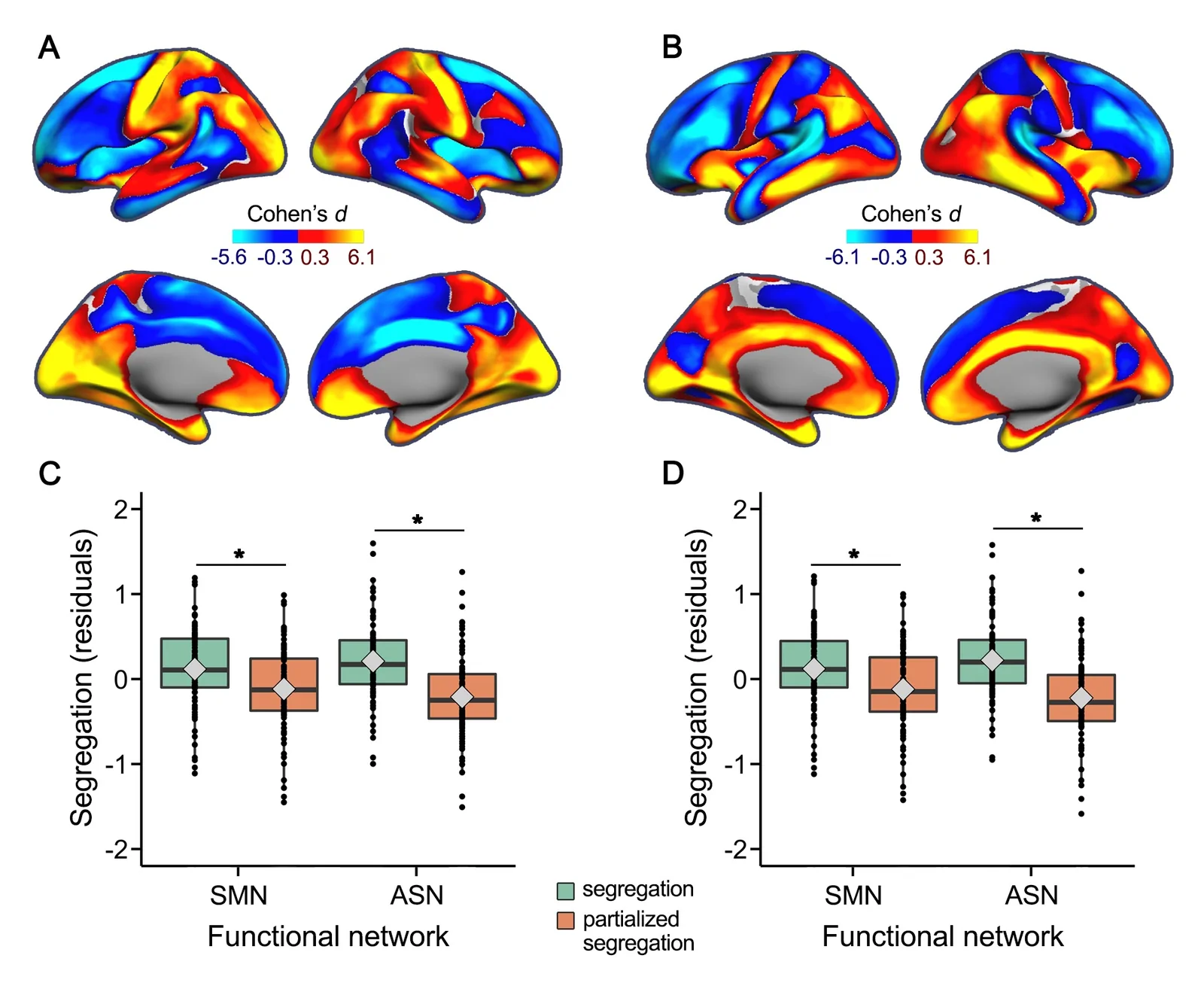
**Mediating role of GM-Aβ load coupling on network segregation**. (**A**) Group-level results of a one-sample t-test performed on individual coupling maps between cortical thickness and cortical Aβ load (FBB-PET), adjusted for central obesity, HOMA-IR, and covariates (age, sex, education years, APOE4, and overall obesity). Lateral and medial views of the left and right hemispheres display effect sizes (Cohen’s d), with warm colors indicating positive associations and cool colors negative associations. (**B**) Same analysis as in A, but with intracortical myelin as the predictor. (**C**) Network segregation for sensorimotor (SMN) and associative networks (ASN) before and after adjusting FC for residuals of the individual-level coupling between cortical thickness and cortical Aβ accumulation, while controlling for central obesity, HOMA-IR, and covariates. Significant differences are marked with asterisks (p_FWE_ < 0.05). (**D**) Same as C, but adjusting FC for residuals of the individual-level coupling between intracortical myelin and cortical Aβ accumulation. Plots in panels C and D include individual subject data points, medians, means (grey diamonds), interquartile ranges (boxes), and whiskers; outliers outside the whiskers are indicated when present.

### Multiple mediators: HOMA-IR → GM structure → FDG-PET ↔ FBB-PET

Lastly, we evaluated whether the relationship among HOMA-IR, GM structure, cortical glucose uptake, and cortical Aβ load mediated the association between central obesity and network segregation. For this analysis, we computed four couplings maps for each individual: cortical glucose uptake as the independent variable and cortical Aβ load as the dependent variable, and *vice versa*, each analyzed twice by including either cortical thickness or intracortical myelin as covariates. While the results were similar across analyses, we chose to illustrate the results derived from the FBB→FDG direction in Figure 5 (panels A and B), as this direction showed a significant mediating effect on both SMN and ASN. The results for the opposite direction, which only mediated the ASN, are displayed in Supplementary Figure S2A-B (Appendix 6). Mixed-effects models identified significant differences in network segregation before and after excluding the residuals of the coupling between imaging modalities from FC. Specifically, after adjusting for cortical thickness, cortical glucose uptake, and cortical Aβ load, we observed a significant reduction in segregation for the SMN (F_1,168_ = 891.8, p_FWE_ < 10^-68^, f^2^ = 10.2^L^ [3.5 21.0]) and the ASN (F_1,168_ = 967.7, p_FWE_ < 10^-70^, f^2^ = 11.6^L^ [6.8 19.9]). Similar reductions were found with intracortical myelin as a mediator, with significant decreases in segregation for the SMN (F_1,168_ = 884.8, p_FWE_ < 10^-68^, f^2^ = 10.1^L^ [3.4 21.1]) and ASN (F_1,168_ = 968.1, p_FWE_ < 10^-70^, f^2^ = 11.6^L^ [6.42 0.9]). These results are illustrated in Figure 5C-D.

**Figure 5.**
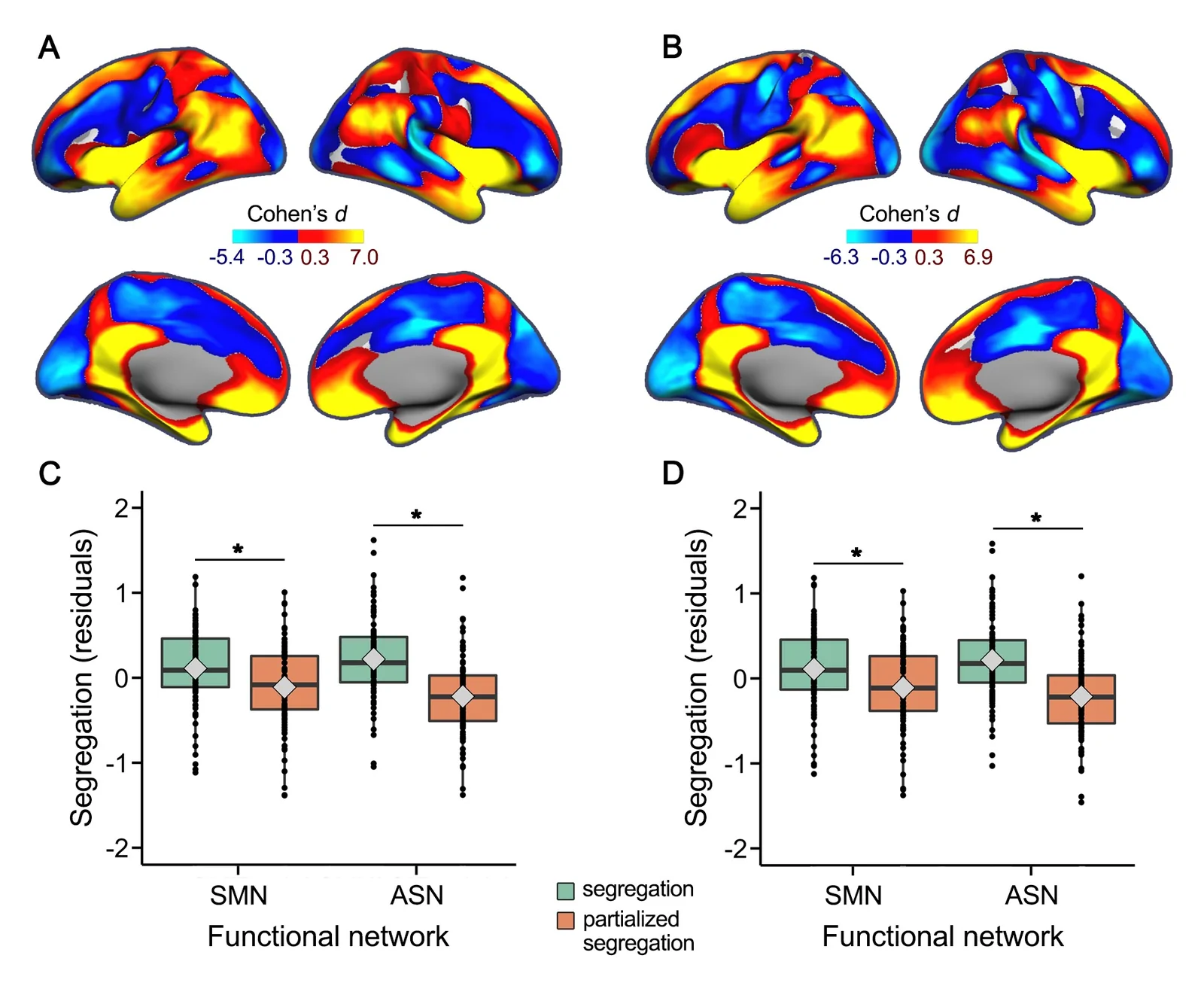
**Mediating role of GM-Aβ-FDG coupling on network segregation**. (**A**) Group-level map of the spatial association between cortical Aβ load (FBB-PET) and cortical glucose uptake (FDG-PET), derived from individual coupling maps in which local variation in cortical thickness was already accounted for. Colors indicate positive (warm) or negative (cool) effects (p_FWE_ < 0.05) after controlling for central obesity, HOMA-IR, and covariates. (**B**) Same analysis as in A, but with intracortical myelin controlled in the individual coupling maps. (**C**) Network segregation of sensorimotor (SMN) and associative networks (ASN) before and after removing variance explained by the coupling shown in A, adjusting for central obesity, HOMA-IR, and covariates. (**D**) Same as C, using the coupling from B. Asterisks mark significant differences (p_FWE_ < 0.05). Bar plots show individual data points, medians, means (grey diamonds), interquartile ranges, and whiskers, with outliers outside the whiskers indicated when present.

Significant differences were also observed after reversing the direction of the coupling. When cortical thickness was included as a covariate, a significant reduction in segregation was found for both the SMN (F_1,168_ = 455.2, p_FWE_ < 10^-48^, f^2^ = 5.2^L^ [1 13.6]) and ASN (F_1,168_ = 1322.9, p_FWE_ < 10^-80^, f^2^ = 15.8^L^ [9.7 24.8]). Similarly, when intracortical myelin was used as a covariate, significant decreases in segregation were observed for the SMN (F_1,168_ = 462.1, p_FWE_ < 10^-49^, f^2^ = 5.3^L^ [0.8 16.9]) and ASN (F_1,168_ = 965.7, p_FWE_ < 10^-70^, f^2^ = 11.5^L^ [7.6 18.2]). These results are shown in Supplementary Figure 2C-D (Appendix 6).

Pathway analyses revealed that the relationship between HOMA-IR, cortical GM structure, FDG-PET, and FBB-PET mediated the effect of central obesity on network segregation in different ways depending on the direction of the coupling. When the coupling was in the FDG→FBB direction, mediation was observed only for ASN. The association of central obesity with SMN segregation persisted after adjusting for all mediators, regardless of whether cortical thickness (F_1,80_ = 3.9, p_FWE_ = 0.05, f^2^ = 0.05^S^ [0.0005 0.21]) or intracortical myelin (F_1,80_ = 4.0, p_FWE_ = 0.048, f^2^ = 0.05^S^ [0.0006 0.22]) was included as mediator. However, when the coupling was reversed (FBB→FDG), mediation effects on segregation were observed for both ASN and SMN.

## DISCUSSION

Our findings underscore the potential role of central obesity in age-related disruptions of brain network segregation, a process essential for maintaining functional specialization within brain systems. Unlike overall obesity, central obesity was associated with decreased network segregation, an effect partially mediated by insulin resistance and linked to alterations in cortical myelin and glucose metabolism. Although cortical Aβ burden was not directly associated with central obesity in our sample, its known interplay with cortical glucose uptake may create a bidirectional feedback loop, whereby impaired glucose metabolism can exacerbate amyloid-related neural dysfunction, and conversely, amyloid pathology may further compromise cortical metabolic function, amplifying disruptions in network segregation. Together, these results highlight the complex interplay between central adiposity, metabolic dysfunction, and cortical GM integrity, suggesting that these factors may collectively influence the organization of functional networks in aging. To summarize these mechanistic relationships, Figure 6 provides a graphical representation of the proposed pathways linking central obesity to disrupted brain network segregation.

**Figure 6.**
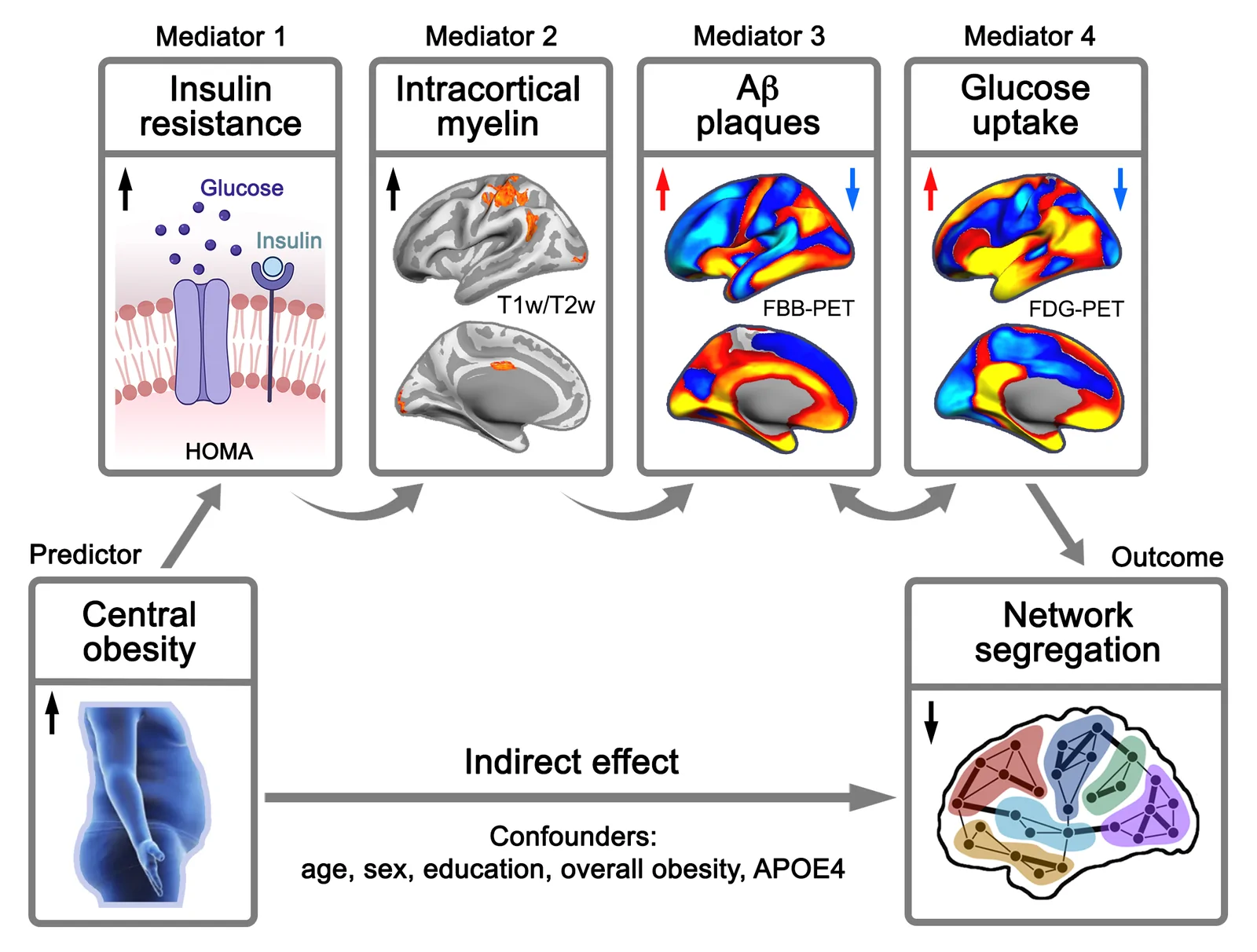
**Proposed mechanistic model linking central obesity to reduced brain network segregation**. This graphical summary illustrates the pathways through which central adiposity may impair functional brain organization in aging. Central obesity is associated with insulin resistance, alterations in intracortical myelin, and changes in cortical glucose metabolism, which in turn contribute to decreased network segregation. Although cortical Aβ burden was not directly associated with central obesity in our sample, its interaction with glucose metabolism may create a bidirectional feedback loop, further amplifying disruptions in network segregation. Together, these interconnected mechanisms highlight the role of metabolic dysfunction as a key mediator linking central adiposity to compromised functional specialization in brain networks.

Results indicated that central obesity, independent of overall obesity, was the primary contributor to impairments in brain network segregation. In our sample, men represented a substantial proportion of individuals with central adiposity. However, exploratory analyses revealed no significant interactions between sex and central obesity in relation to HOMA-IR or network segregation. This suggests that, in our sample, the associations observed are not clearly moderated by sex at these initial steps of the pathway. Nonetheless, previous studies highlight the complexity of sex-specific effects. While men often show stronger associations between central obesity and insulin resistance [74,75], women may be more susceptible to certain brain structural changes, such as reductions in GM volume [5]. Interactions between adipose tissue type and sex further modulate these relationships, with sex-specific effects detected for subcutaneous abdominal fat but not visceral fat [6], and associations between visceral fat and cortical thickness differing across quintiles and in opposite directions for men and women [8]. Hormonal fluctuations [76] and differences in fat distribution [77] likely contribute to these divergent outcomes. Therefore, although our main findings remained significant after adjusting for sex and no moderation was detected in the current sample, caution is warranted in generalizing these results. Future studies with larger and more balanced cohorts will be essential to investigate potential sex-specific effects more comprehensively.

The stronger association of central obesity with brain network segregation, compared to overall obesity, may be linked to alterations in cortical GM structure that appear to occur independently of underlying AD pathology, as suggested by previous findings [78]. However, cortical thickness itself did not appear to account for the observed association with network segregation in the present study. Rather, insulin resistance emerged as a key factor associated with reduced network segregation, suggesting a possible pathway through which central adiposity impacts functional brain organization. This is consistent with previous research showing that in T2D, insulin resistance correlates with reduced FC in cognition-related regions, such as between the posterior cingulate cortex and middle temporal gyrus [79], and that in overall obesity, it disrupts reward-processing networks [80]. Importantly, insulin resistance was not a direct mediator of the association between central obesity and network segregation. Rather, it was its relationship with intracortical myelin in specific cortical regions that, in turn, was associated with reduced network segregation. These observations highlight the potential relevance of intracortical myelin in linking metabolic alterations to functional network organization. Myelinated axons in GM are critical for efficient cortical communication [81] and breakdown of cortical myelin have been associated with cognitive impairment in conditions such as APOE ε4 carriers [47,82], autism [83], and schizophrenia [84]. In our study, higher HOMA-IR was associated with increased intracortical myelin content in specific cortical regions, suggesting a compensatory response to metabolic stress. While previous studies reported bidirectional changes in intracortical myelin associated with AD pathology [82,85] and BMI [44], our results align specifically with the positive direction, indicating that insulin resistance associated with central obesity may drive region-specific myelin increases. These patterns may reflect adaptive remodeling of myelin under metabolic stress, potentially influenced by inflammatory mechanisms [86], while at a regional level, higher myelin content could also indicate abnormal remyelination processes associated with Aβ burden, characterized by increased oligodendrogenesis and thicker myelin sheaths [87,88].

Notably, the relationship between cortical glucose uptake and Aβ load exhibited direction-dependent mediation effects on network segregation. When cortical glucose uptake preceded Aβ load, the effect was restricted to associative networks. However, when Aβ load preceded glucose metabolism, mediation extended to sensorimotor networks. These findings suggest that the temporal order of these events plays a crucial role in determining the brain networks affected, with Aβ deposition having a broader impact when it precedes metabolic changes. This aligns with the ongoing debate on the temporal dynamics of Aβ deposition and cortical hypometabolism in AD. Some studies have suggested that Aβ pathology precedes metabolic disruptions and drives neurodegeneration, while others propose that metabolic dysfunction contributes to Aβ accumulation [89]. Our results indicate that when Aβ load precedes metabolic changes, it leads to more widespread disruption, affecting both associative and sensorimotor networks, likely due to the neurotoxic effects of Aβ. In contrast, when metabolic dysfunction precedes Aβ deposition, the effects are more confined to associative networks, involving tau-prone regions more susceptible to neurodegeneration. These findings contribute to the broader understanding of the complex interplay between Aβ pathology and metabolic dysfunction, where one process may amplify the other, leading to more pronounced disruptions in brain networks as AD progresses.

Finally, our study raises important questions about the potential mechanisms underlying disruptions in network segregation. Both cortical glucose metabolism and Aβ burden were found to affect FC, though likely through different pathways. Disruptions in cortical glucose metabolism, driven by insulin resistance may compromise cortical GM integrity, which in turn could impair neuronal and glial function, potentially leading to reduced dendritic spine density, decreased excitatory neurotransmission, and impaired hippocampal long-term potentiation [90]. Aβ oligomers, in contrast, disrupt synaptic plasticity and neuronal functions, possibly through their toxic effects on synapses and glial cells [91,92]. Although Aβ plaques themselves may not directly impair synaptic activity, they could influence the levels of toxic oligomers, which in turn affect synaptic function [93]. Collectively, these processes contribute to macroscopic disruptions in functional brain networks, with Aβ pathology potentially exacerbating the effects of metabolic dysfunction and further diminishing functional network integrity, which may underlie deficits in cognitive efficiency and adaptive information processing [85,94-96]. While systemic inflammation and vascular risk factors may also contribute to the impact of central obesity on network segregation or modulate the relationships among the mediators considered here, these mechanisms were not directly measured in our study. This represents a limitation, meaning that our findings cannot account for their potential influence. Consequently, our discussion focuses primarily on the metabolic pathway, leaving the precise role of other potential mechanisms to be explored in future work.

In addition to the limitation noted above regarding the lack of systemic inflammation and vascular measures, there are several other considerations in this study. First, the relatively small sample size limits the generalizability of our findings and increases the risk of both Type I and Type II errors. Given the high-dimensional, vertex-wise nature of our analyses, the moderate sample size limits sensitivity to detect small effects; non-significant results may reflect insufficient power rather than absence of associations, and the observed effects likely represent the more robust signals detectable in this sample. For instance, correlations smaller than ~0.20 might not reach significance, while medium-to-large effects (r ≥ 0.30) would be detectable. Importantly, our main finding that central, rather than overall, obesity is associated with reduced network segregation remained robust even after adjusting for overall obesity, suggesting that this result is unlikely to be influenced by power limitations. Second, the cross-sectional design precludes causal inference. Although our mediation analyses explored plausible sequential pathways linking insulin resistance, GM structure, cortical glucose uptake, and Aβ load, the models did not use standard bootstrapping to estimate indirect effects due to computational constraints. Consequently, the analyses identify associations consistent with mediation but cannot formally quantify indirect effects or test their significance in the usual manner. Third, while sex was included as a covariate and exploratory interaction analyses were performed, the limited sample size and imbalance reduced statistical power, constraining our ability to detect potential sex-specific effects. Finally, our sample was relatively homogeneous in terms of ethnic and racial background and consisted of generally healthy volunteers. As a result, caution is warranted when generalizing these findings to broader or more diverse populations, including individuals with different demographic, cultural, or health characteristics. Future studies with larger and more representative samples are needed to confirm the robustness and generalizability of the observed associations.

Nevertheless, this study has also noteworthy strengths. To our knowledge, it is the first to explore the distinct effects of central versus overall obesity on brain network segregation, offering valuable insights into how these two phenotypes independently affect brain function in aging. Furthermore, our sequential analysis of potential mediators employed advanced imaging techniques and cutting-edge analytical methods, including cross-modality coupling at the individual level. Lastly, by analyzing network segregation at the individual node level, we adopted an innovative approach that enabled the correlation of BOLD signal data across nodes, the inclusion of imaging modalities as covariates, and the application of mixed models to account for group-level variables. These strengths collectively establish a solid foundation for understanding the intricate relationships between central obesity, metabolic dysfunction, cortical GM structure, and Aβ pathology in aging.

In summary, our study highlights the significant impact of central obesity on brain network segregation during aging, with insulin resistance emerging as a key mediator. We identified a cascade of neurobiological changes, including cortical alterations in myelin content, glucose uptake, and Aβ accumulation, which together disrupt network segregation, ultimately diminishing functional specialization. These findings provide important insights into how metabolic dysfunction can influence brain health, underscoring the need to address central obesity and insulin resistance as modifiable risk factors to preserve both network segregation and overall functional network integrity in aging populations. Consistent with our observations, a recent longitudinal intervention study reported that improving insulin sensitivity helped preserve cerebral glucose uptake, FC, brain volume, and cognition in insulin-resistant older adults [23]. Targeting these conditions may therefore offer a promising avenue for preserving cognitive function and reducing the burden of age-related neurodegenerative decline in the general population.

## CRediT authorship contribution statement

Marina Fernandez-Alvarez: Conceptualization, Data curation, Formal analysis, Investigation, Software, Visualization, Writing - review and editing. Karel M. Lopez-Vilaret: Data curation, Formal analysis, Software, Writing - review and editing. Jose L. Cantero: Conceptualization, Methodology, Resources, Writing - review and editing. Mercedes Atienza: Conceptualization, Formal analysis, Methodology, Resources, Supervision, Visualization, Writing - original draft, Writing - review and editing.

## Supplementary Materials

The Supplementary data can be found online at: www.aginganddisease.org/EN/10.14336/AD.2025.0887.

## Data Availability

Anonymized data are available from the corresponding author upon reasonable request. The analysis code relies on a specialized software environment with specific hardware requirements and cannot be shared in a fully executable form; however, portions of the code and guidance for reproducing key analyses can be provided upon request.
