# Supplementary material for: Central Obesity Disrupts Brain Network Organization in Aging via Metabolic and Structural Pathways
Source: Aging Dis. 2026 Oct 16;17(6):3380–400. doi: 10.14336/AD.2025.0887 (PMC13653706; doi:10.14336/AD.2025.0887)
Supplement: Supplementary file 1 [file AD-17-6-3380-s.pdf]

SUPPLEMENTARY DATA

Central Obesity Disrupts Brain Network Organization in Aging via Metabolic and Structural Pathways

Marina Fernandez-Alvarez, Karel M. Lopez-Vilaret, Jose L. Cantero, Mercedes Atienza

© 2025. Fernandez-Alvarez M et al. Published online at http://www.aginganddisease.org/EN/10.14336/AD.2025.0887

SUPPLEMENTARY DATA

Appendix 1

MRI preprocessing, cortical thickness estimation, and cortical T1w/T2w ratio map generation

Structural MRI data were processed using Freesurfer v6.0 software (https://surfer.nmr.mgh.harvard.edu/), following a pipeline that involved different steps. Initially, brain extraction was performed, followed by automated tissue segmentation and the creation of cortical surfaces for both WM and pial regions. The surfaces were further processed to correct topological errors and to inflate the cortical mantle. To standardize the processing, the pial surfaces were mapped to a spherical coordinate system [1]. In cases of misplacement in the pial surface or incorrect segmentation of WM, manual corrections were made by trained technicians. Cortical thickness was then determined by calculating the average of the shortest distances from the pial surface to the GM-WM boundary at each vertex along the cortical mantle.

For co-registration of T2w to T1w images, we applied the *bbregister* tool with trilinear interpolation and used a boundary-based cost function, constraining the transformations to 6 degrees of freedom [2]. To correct for intensity inhomogeneities, the *N4BiasFieldCorrection* algorithm was applied separately to both T1w and T2w images [3]. The bias-corrected T1w/T2w ratio volumes were calculated at the midpoint between the GM and WM surfaces. To mitigate the contamination of GM signal by surrounding WM and cerebrospinal fluid, we used the geometric transfer matrix method implemented in PETsurfer to adjust the T1w/T2w ratio maps in a region-based voxel-wise manner [4].

For each participant, cortical maps from all imaging modalities were projected onto an average cortical surface using 7th, 6th, and 5th order icosahedron tessellations, corresponding to 163,842, 40,960, and 15,242 vertices, respectively (*mri_vol2surf*.) The 7th order tessellation was utilized to investigate the effects of obesity and insulin resistance on cortical GM measurements at the vertex level. The 6th order tessellation, which closely aligns with the surface used in cortical parcellation [5], was employed to analyze network segregation. The 5th order tessellation was used to assess the coupling between imaging modalities and evaluate the impact of different regressors at the vertex level [6]. For denoising, a spherical wavelet approach was applied to the 7th and 5th order tessellation surfaces [7], while a small Gaussian kernel with a full width at half maximum (FWHM) of 2 mm was applied to 6th order tessellation surfaces for parcellation-based analyses.

References

1. Fischl B, Dale AM (2000). Measuring the thickness of the human cerebral cortex from magnetic resonance images. Proc Natl Acad Sci U S A, 97:11050-11055.
2. Greve DN, Fischl B (2009). Accurate and robust brain image alignment using boundary-based registration. Neuroimage, 48:63-72.
3. Tustison NJ, Avants BB, Cook PA, Zheng Y, Egan A, Yushkevich PA, et al. (2010). N4ITK: improved N3 bias correction. IEEE Trans Med Imaging, 29:1310-1320.
4. Greve DN, Salat DH, Bowen SL, Izquierdo-Garcia D, Schultz AP, Catana C, et al. (2016). Different partial volume correction methods lead to different conclusions: An (18)F-FDG-PET study of aging. Neuroimage, 132:334-343.
5. Gordon EM, Laumann TO, Adeyemo B, Huckins JF, Kelley WM, Petersen SE (2016). Generation and Evaluation of a Cortical Area Parcellation from Resting-State Correlations. Cereb Cortex, 26:288-303.

© 2025. Fernandez-Alvarez M et al. Published online at http://www.aginganddisease.org/EN/10.14336/AD.2025.0887

6. Vandekar SN, Shinohara RT, Raznahan A, Hopson RD, Roalf DR, Ruparel K, et al. (2016). Subject-level measurement of local cortical coupling. Neuroimage, 133:88-97.
7. Bernal-Rusiel JL, Atienza M, Cantero JL (2008). Detection of focal changes in human cortical thickness: spherical wavelets versus Gaussian smoothing. Neuroimage, 41:1278-1292.

Appendix 2

fMRI preprocessing

Resting-state fMRI data preprocessing was conducted in individual subject space using the AFNI software v20.3.03 (https://afni.nimh.nih.gov/). This included removal of high-frequency spikes (*3dDespike*), application of RETROICOR to reduce motion-related artifacts from cardiac and respiratory cycles [1], and correction for slice acquisition time (*3dTshift*). Rigid body motion correction was applied to align the EPI scans to the first volume (*3dVolreg*) and then, the aligned EPI scans were co-registered with the corresponding T1w images (*align_epi_anat.py*; cost function: lpc+ZZ). Nuisance regressors, including head motion parameters, physiological fluctuations (e.g., pulse and respiration), and their derivatives (from both head motion and physiological signals), as well as WM and CSF signal regressors, were removed through multiple regression, and a band-pass filter (0.01-0.1 Hz) was applied simultaneously (*3dTproject*). Finally, preprocessed resting-state fMRI scans were projected onto the average cortical surface.

Quality control was an integral part of the preprocessing pipeline. Motion-related outliers were excluded when the Euclidean norm (*enorm*) threshold exceeded 0.1 mm in head motion. When more than 3% of voxels exhibited signal intensities deviating from the median absolute deviation of the time series (*3dToutcount*), the corresponding dynamics were removed. After applying censoring, none of the participants exhibited more than 20% artifactual dynamics. EPI-to-T1w registration accuracy was visually inspected, and physiological data alignment was confirmed to ensure proper synchronization with the brain data. The effectiveness of RETROICOR in correcting physiological noise was also checked by observing improved signal differentiation, particularly in ventricular regions, with poorly corrected cases flagged as outliers.

References

1. Glover GH, Li TQ, Ress D (2000). Image-based method for retrospective correction of physiological motion effects in fMRI: RETROICOR. Magn Reson Med, 44:162-167.

Appendix 3

PET data acquisition and preprocessing

PET imaging was conducted using a Philips Gemini 16 PET/CT scanner (Philips, Best, Netherlands). Data were adjusted for radioactive decay, dead time, attenuation, and scatter. Reconstructed images achieved an isotropic voxel resolution of 2 mm^3 through iterative processing.

Participants fasted for 8 hours before the FDG-PET session. The procedure involved administering approximately 185 MBq of FDG in a calm, low-light setting. Imaging began 30 minutes after injection and lasted 10 minutes. For Aβ detection, participants received approximately 300 MBq of FBB

© 2025. Fernandez-Alvarez M et al. Published online at http://www.aginganddisease.org/EN/10.14336/AD.2025.0887

(NeuraCeq, Piramal Pharma) 90 minutes before scanning. The FBB-PET procedure lasted 20 minutes, divided into four 5-minute dynamic frames. Motion artifacts were evaluated in each frame, with no significant head movement observed. Therefore, all dynamic frames were combined into a single static image for quantitative assessment.

To correct for partial volume effects, we used PETSurfer, a toolbox within Freesurfer v6.0 (https://surfer.nmr.mgh.harvard.edu/). Correction used the region-based voxel-wise approach, assuming a uniform point spread function of 6 mm. The SUVR for each vertex was calculated by dividing the mean corrected uptake at that vertex by that in the reference structure (pons structure for FDG-PET data and cerebellar GM for FBB-PET data). Preprocessed PET data were projected onto the average cortical surfaces.

Appendix 4

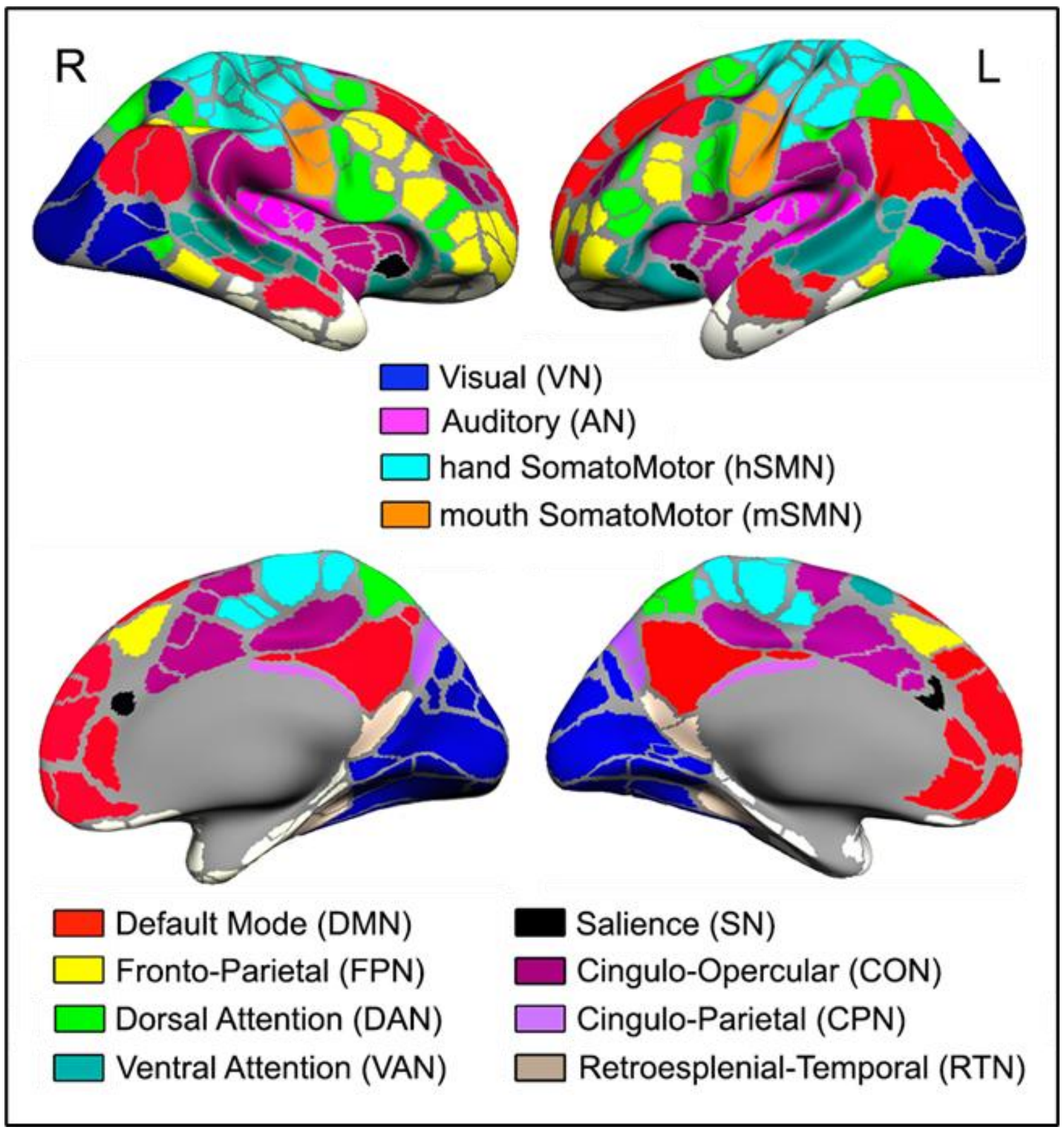

Supplementary Figure 1. Parcellation scheme. Cortical parcels and functional networks included in the Gordon parcellation scheme [5]. R: right; L: left.

SUPPLEMENTARY DATA

Appendix 5

Supplementary Table 1. Demographic and metabolic characteristics of participants by overall obesity category, with data stratified by sex (male = 31; female = 58).

	Overall obesity					
**Variable**	**Normal**		**Overweight**		**Obesity**	
Sex	**F**	**M**	**F**	**M**	**F**	**M**
Participants	22	8	22	15	14	8
Age	65.4 ± 6.5	68.8 ± 6.5	65.5 ± 5.9	64.7 ± 5.6	66.9 ± 6.5	64.1 ± 8.7
Education years	12 ± 5.1	9.6 ± 5.7	11.8 ± 5.3	12.3 ± 3.7	8.1 ± 4.0	11.0 ± 4.7
APOE4 (%)	4 (18.2)	2 (25.0)	4 (18.2)	7 (46.7)	3 (21.4)	1 (6.7)
BMI*	21.5 ± 1.6	23.4 ± 0.8*	27.0 ± 1.3	27.0 ± 1.5	32.8 ± 2.6	33.6 ± 3.9
Waist circumference (cm)	76.4 ± 5.5	90.2 ± 3.9	86.7 ± 6.7	96.9 ± 5.1	100.0 ± 7.9	111.7 ± 7.2
Glucose (mmol/l)	4.9 ± 0.5	5.1 ± 1.0	5.1 ± 0.9	5.4 ± 0.9	5.4 ± 0.6	5.5 ± 1.0
Insulin (mU/l)	4.4 ± 2.0	6.0 ± 2.7	7.6 ± 4.3	7.3 ± 3.3	11.1 ± 5.8	16.7 ± 6.9
HOMA-IR	1.0 ± 0.5	1.4 ± 0.9	1.8 ± 1.3	1.8 ± 1.1	2.8 ± 1.6	4.0 ± 1.6
HbA1c (%)	5.3 ± 0.5	5.8 ± 0.5	5.3 ± 0.7	5.7 ± 0.6	5.6 ± 0.8	5.9 ± 0.8
Hypertension (%)	9 (40.9)	3 (37.5)	8 (36.4)	10 (66.7)	8 (57.1)	5 (62.5)
Dyslipidemia (%)	4 (18.2)	1 (12.5)	2 (9.1)	2 (13.3)	3 (21.4)	3 (37.5)
Type 2 diabetes (%)	0 (0)	1 (12.5)	1 (4.5)	1 (6.7)	2 (14.3)	1 (12.5)
Metabolic syndrome (%)	0 (0)	1 (12.5)	2 (9.1)	2 (13.3)	4 (28.6)	3 (37.5)

Note. Continuous variables are shown as mean ± SD. Categorical variables are shown as n (%) within each obesity category. BMI = body mass index; HbA1c = glycoside hemoglobin

Supplementary Table 2. Demographic and metabolic characteristics of participants by central obesity category, with data stratified by sex (male = 31; female = 58).

	Central obesity			
**Variable**	**No**		**Yes**	
Sex	**F**	**M**	**F**	**M**
Participants	37	12	21	19
Age*	65.4 ± 6.1	68.8 ± 5.7	66.5 ± 6.5	63.6 ± 6.7*
Education years*	12.5 ± 5.3	9.8 ± 5.3	8.1 ± 3.4	12.2 ± 3.8*
APOE4 (%)	6 (13.5)	4 (33.3)	5 (23.8)	6 (31.6)
BMI	23.7 ± 3.0	24.4 ± 1.7	31.0 ± 3.4	29.9 ± 4.2
Waist circumference (cm)*	79.1 ± 6.0	90.1 ± 2.8	98.1 ± 7.4	104.6 ± 8.1*
Glucose (mmol/l)	4.9 ± 0.5	5.0 ± 0.7	5.4 ± 0.9	5.6 ± 1.0
Insulin (mU/l)	5.0 ± 2.4	6.0 ± 2.6	11.2 ± 5.4	11.5 ± 6.8
HOMA-IR	1.1 ± 0.6	1.4 ± 0.7	2.8 ± 1.6	2.9 ± 1.7
HbA1c (%)	5.3 ± 0.5	5.8 ± 0.4	5.5 ± 0.9	5.8 ± 0.7
Hypertension (%)	15 (40.5)	6 (50.0)	10 (47.6)	12 (63.2)
Dyslipidemia (%)	5 (13.5)	3 (25.0)	4 (19.0)	3 (15.8)
Type 2 diabetes (%)	0 (0)	1 (8.3)	3 (14.3)	2 (10.5)
Metabolic syndrome (%)	0 (0)	0 (0)	6 (28.6)	6 (31.6)

Note. Continuous variables are shown as mean ± SD. Categorical variables are shown as n (%) within each obesity category. BMI = body mass index; HbA1c = glycoside hemoglobin.

© 2025. Fernandez-Alvarez M et al. Published online at http://www.aginganddisease.org/EN/10.14336/AD.2025.0887

Supplementary Table 3. Summary of adjusted linear models examining the association of overall and central obesity with different anthropometric and metabolic variables.

DV	Regressor	F_{df}	*p*-value	Size effect (f^2)	*95% IC*	*Adj. R^2*
BMI	Overall Ob	$F_{1,82}$ = 226.3	10^{33}	5.5^L	3.1 – 7.3	0.85
	Central Ob	$F_{1,83}$ = 80.8	10^{13}	1.0^L	0.6 – 1.5	0.51
Waist circumference	Overall Ob	$F_{1,82}$ = 77.9	10^{18}	1.9^L	1.2 – 2.7	0.74
	Central Ob	$F_{1,83}$ = 33.0	10^{6}	1.5^L	1.0 – 2.2	0.71
Insulin	Overall Ob	$F_{1,82}$ = 22.8	10^{7}	0.6^L	0.2 – 0.9	0.35
	Central Ob	$F_{1,83}$ = 33.0	10^{6}	1.5^L	1.0 – 2.2	0.71
Glucose	Overall Ob	$F_{1,82}$ = 3.0	0.05	0.07^S	0.002 – 0.2	0.08
	Central Ob	$F_{1,82}$ = 10.2	0.002	0.12^S	0.01 – 0.3	0.13
HOMA-IR	Overall Ob	$F_{1,82}$ = 20.0	10^{7}	0.5^L	0.2 – 0.9	0.32
	Central Ob	$F_{1,82}$ = 40.4	10^{7}	0.5^L	0.2 – 0.9	0.33
HbA1c	Overall Ob	$F_{1,82}$ = 1.55	0.2	0.04^S	0.0004 – 0.1	0.13
	Central Ob	$F_{1,82}$ = 0.75	0.4	0.009	0 – 0.09	0.11

Note: linear models were adjusted by age, sex, education and APOE4. BMI = body mass index; HbA1c = glycoside hemoglobin; Ob = obesity.

Supplementary Table 4. Mean (±SD) neuropsychological test scores across cognitive domains and subcomponents for participants grouped by overall and central obesity status, with group comparisons adjusted for age, sex, education years, and APOE4 status.

**Cognitive domain**<br>Cognitive process	TEST (score)	**Overall obesity**			**Central obesity**	
		**Normal**	**Overweight**	**Obesity**	**No**	**Yes**
**Global cognition**	MMSE	28.9 (1.3)	29.0 (1.1)	28.3 (1.8)	28.9 (1.3)	28.7 (1.5)
**Episodic memory**						
Immediate recall without cue	MBT (TFR)	14.6 (4.2)	14.4 (5.5)	13.9 (5.4)	15.2 (4.8)	13.3 (5.2)
Immediate recall with cue	MBT (TPR)	23.1 (4.4)	23.8 (5.1)	22.0 (5.8)	23.7 (4.7)	22.4 (5.4)
Delayed recall without cue	MBT (TDFR)	15.2 (4.8)	15.1 (5.9)	13.2 (5.6)	15.8 (5.4)	13.3 (5.4)
Delayed recall with cue	MBT (TDPR)	22.2 (5.1)	22.9 (5.6)	21.2 (7.0)	22.9 (5.4)	21.4 (6.2)
**Associative memory**						
Immediate	S-FNAME (CRN+CRO)	9.5 (5.3)	9.4 (4.2)	9.6 (5.8)	9.8 (4.9)	9.2 (4.9)
Delayed	S-FNAME (CRN30+CRO30)	9.0 (5.1)	8.9 (4.0)	9.0 (5.6)	9.2 (4.8)	8. (4.7)
**Attention**						
Selective attention	D2 (C)	1.4 (3.7)	1.3 (3.0)	2.4 (4.4)	1.1 (2.9)	2.2 (4.2)
	D2 (O)	12.8 (9.4)	12.9 (11.4)	16.4 (11.9)	12.9 (9.0)	14.7 (12.9)
	D2 (CON)	133.2 (39.8)	151.3 (42.3)	127.2 (47.4)	139.2 (41.8)	139.3 (46.2)
Sustained attention	D2 (TA)	134.5 (38.6)	152.6 (40.7)	130.0 (43.8)	140.3 (40.8)	141.7 (43.0)
	D2 (VAR)	11.7 (3.4)	10.7 (3.2)	12.6 (4.5)	11.6 (3.2)	11.5 (4.2)
**Processing speed**	D2 (TR)	349.1 (95.5)	392.8 (102.8)	347.4 (101.3)	362.8 (102.8)	371.8 (100.6)
	TMT – A	43.9 (11.8)	40.5 (17.8)	48.2 (21.0)	44.6 (16.3)	42.2 (18.0)
**Working memory**	Letter-Number (WAIS - III)	8.3 (2.0)	**10.1 (2.9)***	8.0 (2.6)	8.9 (2.3)	9.0 (3.1)
**Executive function**						
Mental flexibility	TMT – B	132.6 (72.3)	**98.7 (55.6)***	145.6 (114.7)	123.9 (69.8)	**119.1 (93.5)***
	Semantic verbal fluency	20.3 (7.1)	20.6 (5.0)	19.0 (4.5)	20.3 (6.6)	20.0 (4.5)
	Phonological verbal fluency	15.8 (4.8)	16.6 (5.3)	14.7 (4.9)	15.7 (5.0)	16.0 (5.2)
Planning	ToL (TCS)	3.5 (1.7)	3.7 (2.1)	3.4 (1.7)	3.4 (1.8)	3.7 (1.9)
	ToL (TMS)	38.5 (14.6)	38.0 (16.3)	38.2 (16.1)	38.7 (13.9)	37.6 (17.5)

© 2025. Fernandez-Alvarez M et al. Published online at http://www.aginganddisease.org/EN/10.14336/AD.2025.0887

**Language**						
Naming	BNT	12.1 (2.5)	12.5 (1.7)	12.2 (2.1)	12.1 (2.3)	12.5 (1.8)

Note: BNT: Boston Naming Test; D2 (C): D2 Test of Attention – Concentration Performance; D2 (CON): Concentration Index; D2 (TA): Total Number of Items Processed; D2 (TR): Total Number of Items Attempted; D2 (VAR): Variability of Performance; FCSRT (E1RL): Free and Cued Selective Reminding Test – Early 1st Recall List; MBT (TDFR): Memory Binding Test – Total Delayed Free Recall; MBT (TDPR): Total Delayed Paired Recall; MBT (TPR): Total Paired Recall; MBT (TFR): Total Free Recall; MMSE = Mini-Mental State Examination; S-FNAME (CRN+CRO): Short Form of the Face-Name Associative Memory Exam – Correct Recall Names + Correct Recall Occupations; S-FNAME (CRN30+CRO30): Correct Recall Names + Correct Recall Occupations after 30 min delay; ToL (TCS): Tower of London Test (Time to Complete Solution); ToL (TMS): Tower of London Test (Total Move Score); TMT – A: Trail Making Test – Part A; TMT – B: Trail Making Test – Part B. *p < 0.05, uncorrected for multiple comparisons.

Supplementary Table 5. Mean (±SD) cortical thickness, intracortical myelin, FDG-PET, FBB-PET, and network segregation values for participants grouped by central obesity status and sex.

**Neuroimaging modality**	**Network**	**No central obesity**		**Central obesity**	
		**F**	**M**	**F**	**M**
Cortical thickness	SMN	2.26 ± 0.07	2.17 ± 0.06	2.22 ± 0.09	2.23 ± 0.12
	ASN	2.39 ± 0.06	2.32 ± 0.07	2.36 ± 0.06	2.38 ± 0.08
Myelin content	SMN	0.28 ± 0.05	0.27 ± 0.03	0.27 ± 0.04	0.31 ± 0.04
	ASN	0.24 ± 0.05	0.24 ± 0.03	0.24 ± 0.04	0.26 ± 0.03
FDG-PET	SMN	2.59 ± 0.26	2.49 ± 0.20	2.49 ± 0.25	2.49 ± 0.31
	ASN	2.50 ± 0.27	2.34 ± 0.21	2.40 ± 0.26	2.39 ± 0.32
FBB-PET	SMN	1.11 ± 0.25	1.18 ± 0.27	1.07 ± 0.19	1.09 ± 0.20
	ASN	1.13 ± 0.34	1.27 ± 0.38	1.09 ± 0.27	1.10 ± 0.24
Segregation	SMN	0.49 ± 0.06	0.52 ± 0.07	0.47 ± 0.07	0.48 ± 0.05
	ASN	0.45 ± 0.07	0.46 ± 0.04	0.40 ± 0.09	0.42 ± 0.07

Note: F = female, M = male, SMN = sensorimotor network, ASN = associative network.

© 2025. Fernandez-Alvarez M et al. Published online at http://www.aginganddisease.org/EN/10.14336/AD.2025.0887

Appendix 6

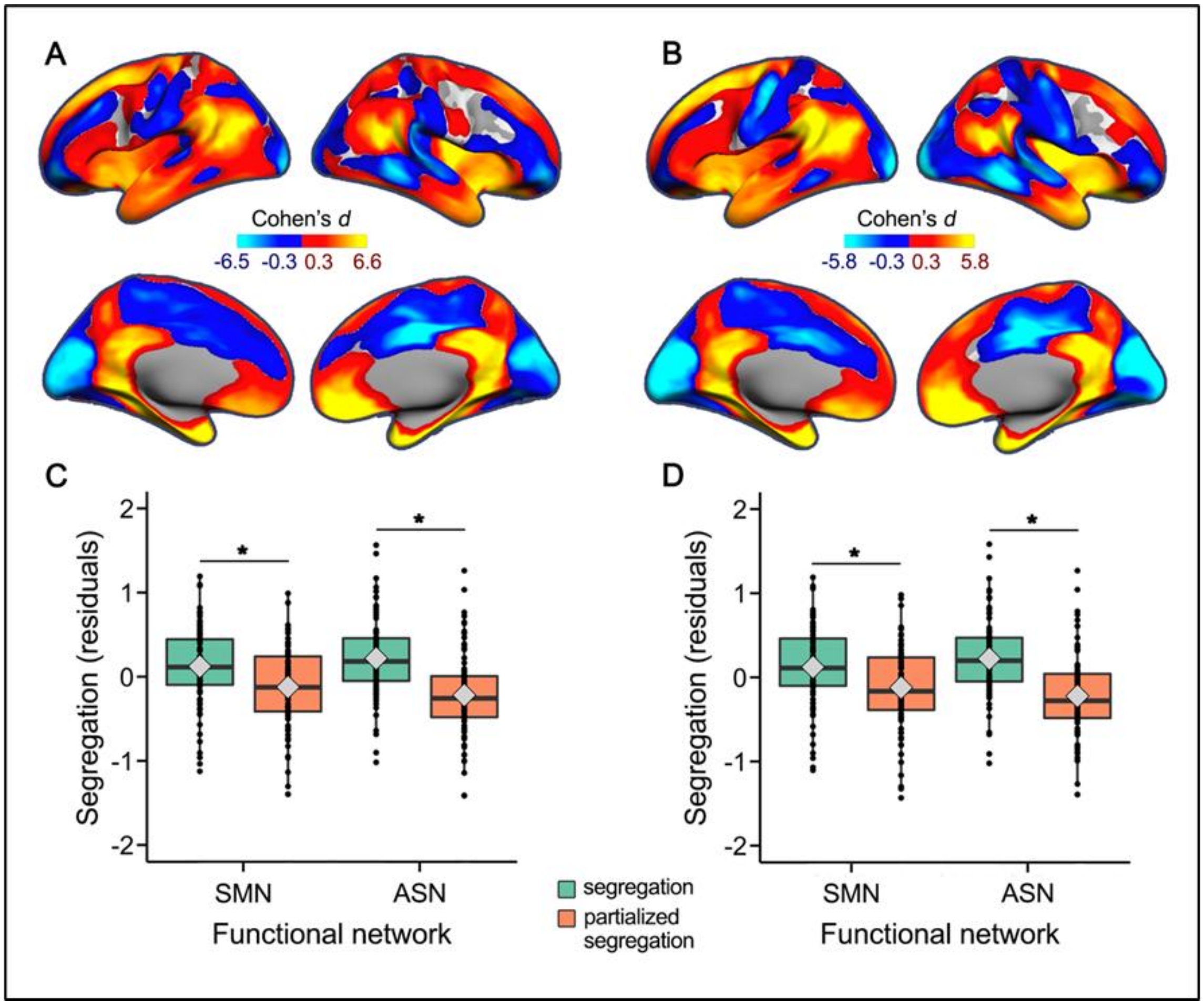

Supplementary Figure 2. Mediating role of GM–FDG–Aβ coupling on network segregation. **A)** Group-level map of the spatial association between cortical glucose uptake (FDG-PET), and cortical Aβ load (FBB-PET) derived from individual coupling maps in which local variation in cortical thickness was already accounted for. Colors indicate positive (warm) or negative (cool) effects ($p_{FWE} < 0.05$) after controlling for central obesity, HOMA-IR, and covariates. **B)** Same analysis as in A, but with intracortical myelin controlled in the individual coupling maps. **C)** Network segregation of sensorimotor (SMN) and associative networks (ASN) before and after removing variance explained by the coupling shown in A, adjusting for central obesity, HOMA-IR, and covariates. **D)** Same as C, using the coupling from B. Asterisks mark significant differences ($p_{FWE} < 0.05$). Bar plots show individual data points, medians, means (grey diamonds), interquartile ranges, and whiskers, with outliers outside the whiskers indicated when present.
